# The metabolic regulator USF-1 is involved in the control of affective behaviour in mice

**DOI:** 10.1038/s41398-022-02266-5

**Published:** 2022-12-01

**Authors:** Spyros Sideromenos, Maria Nikou, Barbara Czuczu, Nikolas Thalheimer, Anna Gundacker, Orsolya Horvath, Laura Cuenca Rico, Peter Stöhrmann, Marco Niello, Timo Partonen, Daniela D. Pollak

**Affiliations:** 1 Department of Neurophysiology and Neuropharmacology, Center for Physiology and Pharmacology, Medical University of Vienna, Schwarzspanierstraße 17, 1090 Vienna, Austria; 2 Institute for Pharmacology, Center for Physiology and Pharmacology, Medical University of Vienna, Schwarzspanierstraße 17, 1090 Vienna, Austria; 3 Mental Health Team, Department of Public Health and Welfare, Finnish Institute for Health and Welfare, Helsinki, Finland

**Keywords:** Molecular neuroscience, Psychiatric disorders

## Abstract

Epidemiological studies indicate a bidirectional association between metabolic disturbances, including obesity and related pathological states, and mood disorders, most prominently major depression. However, the biological mechanisms mediating the comorbid relationship between the deranged metabolic and mood states remain incompletely understood. Here, we tested the hypothesis that the enhanced activation of brown fat tissue (BAT), known to beneficially regulate obesity and accompanying dysfunctional metabolic states, is also paralleled by an alteration of affective behaviour. We used upstream stimulatory factor 1 (USF-1) knock-out (KO) mice as a genetic model of constitutively activated BAT and positive cardiometabolic traits and found a reduction of depression-like and anxiety-like behaviours associated with USF-1 deficiency. Surgical removal of interscapular BAT did not impact the behavioural phenotype of USF-1 KO mice. Further, the absence of USF-1 did not lead to alterations of adult hippocampal neural progenitor cell proliferation, differentiation, or survival. RNA-seq analysis characterised the molecular signature of USF-1 deficiency in the hippocampus and revealed a significant increase in the expression of several members of the X-linked lymphocyte-regulated (xlr) genes, including *xlr3b* and *xlr4b*. Xlr genes are the mouse orthologues of the human FAM9 gene family and are implicated in the regulation of dendritic branching, dendritic spine number and morphology. The transcriptional changes were associated with morphological alterations in hippocampal neurons, manifested in reduced dendritic length and complexity in USF-1 KO mice. Collectively these data suggest that the metabolic regulator USF-1 is involved in the control of affective behaviour in mice and that this modulation of mood states is unrelated to USF-1-dependent BAT activation, but reflected in structural changes in the brain.

## Introduction

The global prevalence of two medical conditions constituting a major burden on public health systems is on unrelenting rise over the last decades: obesity-related cardiometabolic diseases and mood disorders. The escalating epidemic of these non-communicable diseases not only poses a significant socioeconomic problem associated with work disability, high treatment costs and an augmented risk for premature death, but also inflicts a severe load of pain and suffering on the affected individuals and their families. Both conditions are heterogeneous, multifactorial pathological states, not effectively or sufficiently medically controlled in a large percentage of patients, in part due to the still incomplete understanding about the underlying pathogenic mechanisms.

Major depression (MDD), currently worldwide the third-ranked cause of burden of disease and considered to be the top lead by 2030 [1], is often associated with an increased prevalence of several somatic conditions, including obesity and related metabolic diseases [2–5]. On the other hand, patients with an impaired metabolic status also show a higher incidence of MDD [6–9]. This bidirectional association has been quantified for obesity with MDD patients having a 58% increased risk of obesity and obese patients a 55% higher risk of depression [8].

The pathophysiological principles mediating these instances of comorbidity remain largely unrevealed, despite the known role of the brain in controlling food intake and energy homoeostasis [10–12] and the impact of body weight on brain health [13, 14]. Existing hypotheses to explain the bidirectional link between metabolic and mood disorders include a prominent involvement of glucocorticoids, which are tightly involved in the pathogenesis of both conditions [15, 16]. Other possible mediators are key hormonal regulators of metabolic processes, such as leptin and insulin [17–19]. At the cellular levels, alterations in adult hippocampal neurogenesis, closely linked to depression-like behaviour and the therapeutic effects of antidepressant drugs in a wealth of validated animal models (for review, see ref. [20]) are also related to metabolic disorders, including type 1 and type 2 diabetes (for review, see ref. [21]). Last, but not least, dysregulated circadian rhythms have been associated with the development of both mental [22, 23] and metabolic disorders [24, 25], and, interestingly, altered circadian rhythms are connected with impaired biological functions of several possible mediators of the comorbidity of mental and metabolic disorders, including glucocorticoids [26], insulin [27], leptin [28] and adult hippocampal neurogenesis [29, 30].

Since its identification and functional characterisation in adult humans [31, 32], the brown adipose tissue (BAT) has emerged as an important peripheral regulator of energy homoeostasis and metabolism. The inverse correlation between BAT and body weight or glucose levels has made BAT an attractive target in the therapeutic management of metabolic disorders, to enhance insulin sensitivity [33] and to lower the susceptibility for development of obesity [34]. In addition, high BAT activity has been proposed to promote brain health in people [35], but the influence of increased BAT activity on mental health may not be straightforward [36]. Whether an alteration of BAT activity is paralleled by the modulation of depression-like behaviour, has not yet been investigated.

Here, we used USF-1 (upstream stimulatory factor 1)-deficient mice (USF-1 KO) as model to test whether a genetic modification that results in constitutively heightened BAT activity [37] also impacts mood-related behaviours. We show that mice lacking USF-1 display a reduction of depression-like and anxiety-like behaviours in comparison to wildtype (WT) littermates, which persist even after bilateral removal of interscapular BAT depots (iBATx). The decrease in depression-like and anxiety-like behaviours in USF-1 KO mice is not paralleled by a modification of adult hippocampal neurogenesis, but by altered expression of molecular regulators of neuronal morphology and structural adaptations in hippocampal neurons.

This study firstly suggests the transcription factor USF-1 as a molecular hub between cardiometabolic and mental health, with USF-1 deficiency positively affecting both conditions.

## Materials and methods

### Animals

Heterozygous male and female USF-1 deficient mice on C57Bl/6JRccHsd background were obtained from the University of Helsinki [37] and used for breeding of WT and USF-1 KO mice. Mice were weaned at the age of 3 weeks and housed with same-sex littermates. Female and male littermates were used throughout the study. Two weeks before the onset of behavioural experiments, mice were single-housed. Since group-housed animals tend to huddle together they temperature of their immediate surrounding changes in an uncontrolled manner. Considering the temperature-dependent activation of BAT activity it was decided to single-house animals for the present experimental design in order to better regulate the thermal environment of the animals.

Mice were kept at the local animal facility under standard laboratory conditions at a 12:12 h light:dark cycle with food and water available ad libitum. Temperature was kept constant at 22 °C (±1 °C).

All animal experiments were conducted in agreement with the ARRIVE guidelines and the U.K. Animals (Scientific Procedures Act, 1986 and associated guidelines, EU Directive 2010/63/EU for animal experiments) and approved by the national ethical committee on animal care and use (Bundesministerium für Wissenschaft und Forschung: BMBWF-66.009/0175-V/3b/2019).

### Behaviour

#### Forced-swim test (FST)

The FST was carried out using an automated movement tracking software (VideoTrack v3, Viewpoint, Lyon, France) as reported earlier [38]. The test lasted 6 min and the relative immobility (%) of the last 4 min of the test was calculated and used as an index of despair-like behaviour.

#### Novelty suppressed feeding (NSF)

The NSF was conducted according to a published procedure [38]. Mice remained fasted for 24 h before the actual NSF test. Mice that lost more than 20% of their initial body weight during the food deprivation period were not included in the analysis. A food pellet was fixed on a paper and placed on the centre of a brightly illuminated (800 lux) arena filled with bedding. Mice were always placed on the corner of the arena and the latency to grab and start eating the food pellet was manually measured and used as relevant parameter. After the termination of the test, mice were transferred back to their home cage and given access to a food pellet for 5 min. The amount of food consumed in the home-cage environment was used to control for general changes in the appetitive behaviour.

#### Elevated plus maze (EPM)

Anxiety-like behaviour in the EPM was determined as previously described [39] using an automated tracking system (Videotrack v3, Viewpoint, Lyon, France). The light intensity was 50 and 10 lux in the open and closed arms, respectively. The total test duration was 5 min and the % of entries in the open arms (open-arm entries divided by total entries × 100) was calculated.

#### Sucrose preference test (SPT)

SPT was conducted as published previously [40]. Briefly, mice were presented with two bottles, one containing normal tap water and the other 2% sucrose solution. The volume of liquid consumption from each bottle was recorded for 3 h and used for the calculation of sucrose preference [volume of sucrose consumed divided by (volume of sucrose consumed + volume of water consumed) × 100], which was used as an index of hedonic behaviour.

#### Open-field test (OFT)

The OFT was utilised to assess locomotor activity [41]. Mice were placed in a rectangular arena (27.3 × 27.3 cm^2^) that was illuminated (300 lux). Locomotor behaviour was recorded for 30 min with an automated system (Activity monitor, Med Associates, St. Albans, VT, USA) and the total ambulatory distance was calculated.

#### Rotarod (RR)

The RR was employed to evaluate motor coordination [41]. Mice were placed on a rotating drum with the speed gradually increasing from 4 rounds-per-min to 40 rounds-per-min. Every mouse was subjected to RR test for three times with the inter-trial interval set to 30 min. The latency to fall from the rotating drum was automatically recorded (Med Associates, St. Albans, VT, USA). The averaged latency to fall in the 3 trials was calculated and used as an index of motor coordination.

#### Chronic corticosterone (CORT) treatment

A protocol of chronic corticosterone exposure was employed to experimentally induce a depressive-like state. Chronic CORT treatment was conducted according to previously published procedures [42, 43] using corticosterone hemisuccinate (Q1562-000, Steraloids, Newport, R.I, USA).

### Surgical removal of brown adipose tissue

Interscapular BAT (iBAT) was surgical removed in a cohort of female WT and USF-1 KO mice at 8 weeks of age as described [44]. Briefly, mice were deeply anaesthetised with isoflurane (4–4.5% for induction; 1.7–2.5% for maintenance; Forane, Baxter, Deerfield, IL, USA) and a small incision was made along the dorsal midline. iBAT was exposed and carefully removed. For sham-operated controls iBAT was exposed, but not removed. The health of all animals was closely monitored for two days after the surgical procedure and the body weight was measured. None of the mice lost more than 20% of the initial body weight or show signs of discomfort within two days after the surgery.

### Analysis of adult hippocampal neurogenesis

#### Neurogenesis paradigm and brain collection

To evaluate progenitor cell proliferation and the survival of newborn cells, two different protocols of BrdU injections were employed [45]. For the proliferation paradigm, 4 i.p. injections of 50 mg of BrdU (10 ml/kg, Sigma-Aldrich, St. Louis, MO, USA) were administered every 2 h and mice were sacrificed 24 h after the last injection. For the survival paradigm, mice were injected with 50 mg/kg of BrdU (10 ml/kg) twice per day, for 3 days, and sacrificed 14 days after the first injection. To harvest the brains, mice were deeply anaesthetised and transcardially perfused with 4% paraformaldehyde (PFA). Perfused brains were rapidly collected, stored for 24 h in 4% PFA at 4 °C and then kept in 30% sucrose solution for 48 h at 4°C. Brains were frozen in O.C.T. (Tissue-Tek, Fisher Scientific, Hampton, NH, USA) and maintained at −80 °C until further processing.

#### Immunofluorescence-histochemistry

Thirty micrometer coronal brain sections containing the hippocampus were cut on a Leica cryostat (CM1950, Leica, Wetzlar, Germany). Every 10th section was used for the quantification of BrdU-positive cells, according to a previous published procedure [46]. For the proliferation paradigm, free-floating brain sections were incubated with a mouse anti-BrdU antibody (1:300; Bio-Rad AbD Serotec, Kidlington, UK). For the survival paradigm, sections were incubated with a mouse anti-BrdU antibody (1:300; Bio-Rad AbD Serotec, Kidlington, UK) and, additionally, either with a rabbit anti-NeuN antibody (12943, Cell Signaling Technology, Danvers, MA, USA; 1:500) or with a rabbit anti-GFAP (G4546, Sigma-Aldrich, St. Louis, MO, USA; 1:500). Secondary antibodies were 488 goat anti-mouse (Thermofisher Scientific, Waltham, MA, USA; 1:500; proliferation paradigm), 488 goat anti-rabbit (Thermofisher Scientific, Waltham, MA, USA; 1:500; survival paradigm) and 594 goat anti-mouse (Thermofisher Scientific, Waltham, MA, USA; 1:500; survival paradigm). DAPI was used to stain cell nuclei.

Fluorescent pictures were acquired using a Carl-Zeiss Axiovert-Apotome System with the Axiovision software v4.8 (Oberkochen, Germany). For quantification of BrdU-positive cells, NeuN-positive cells and GFAP-positive cells, the acquired images were analysed in ImageJ [47]. The whole-dentate gyrus was selected and the total number of the BrdU-positive cells in the subgranular zone was counted. The number of the counted cells was normalised to the size of the counted area.

### Brain extraction and RNA isolation

Mice were killed by cervical dislocation and hippocampi were rapidly dissected out from the extracted brain and stored at −80 ˚C until further processing. RNA extraction was performed using miRNEasy Mini Kit (217004, Qiagen, Venlo, Netherlands) following the manufacturer’s instructions. Isolated RNA was further processed with the DNA-free™ Kit (Ambion, Austin, TX, USA).

### RNA-Seq and bioinformatic analysis

Samples were quality-checked using a Bioanalyzer (Agilent Technologies, Santa Clara, CA, USA) and showed RNA Integrity Numbers above 7.0. Library preparation and sequencing was performed as previously described [48]. Data were analysed on the Illumina Basespace platform (Illumina, San Diego, CA, USA) using the RNA-Seq alignment app and followed up using STAR aligner [49] and DESeq2 [50]. Raw *p*-values were corrected by applying the Benjamini-Hochberg method and a false discovery rate of 5% to produce *q-*values.

Genes that demonstrated a sufficient basal mean expression (higher than 100) and the magnitude of the expressional change was higher than 20% (+20% for upregulated and −20% for downregulated genes), were chosen for further analysis. Genes that fulfilled the mentioned criteria were sorted by the adjusted p-value and the top 100 were used for further bioinformatic analysis. The online free software Enrichr [51] was used to compute the Gene ontology (GO) molecular function, the GO biological function and the ENCODE and ChEA Consensus TFs from ChIP-X of the 100 most significant DEGs in the hippocampus of USF-1 mice.

The full mRNAseq dataset generated within this study is available online in the NCBI GEO (gene expression omnibus) functional genomics data repository under the accession number GSE204675.

### Quantitative real time-PCR

Nine-hundred nanograms of purified RNA was converted to cDNA with the RevertAid RT Reverse Transcription Kit (K1691, Thermofischer, Waltham, MA, USA). qRT-PCR was performed using the SYBR Green MasterMix (A6002, Promeg, Madison, WI, USA). β-actin was used as a housekeeping gene and the relative expression of the genes of interest was based on the ΔΔC(t) method [46]. The following primer sequences were used: xlr3b (forward: TTGATGCTGGTAGGGAGGACA, reverse: AGAACTTTGTTAGGTGGCTCTTC), xlr4b (forward: GTTGACCACTTCTTGAAAGTCCA, reverse: CAGAGAGTTTTCCAGCCTGTTT), β-actin (forward: ATG GTG GGA ATG GGT CAG AAG, reverse: TCT CCA TGT CGT CCC AGT TG).

### Analysis of neuronal morphology

#### Golgi-Cox staining

The FD Rapid GolgiStain^TM^ Kit (FD NeuroTechnologies, Columbia, MD, USA) was used for the Golgi-Cox staining procedure for neuronal reconstruction of hippocampal neurons in the hippocampus of USF-1 KO and WT mice following the manufacturer’s instructions.

#### Neurolucida reconstruction and analysis

Neurolucida 10 (MBF Bioscience, Williston, VT, USA) was used for the reconstruction of neuronal morphologies in the CA1 area of Golgi-Cox-stained brains as previously described [52]. Briefly, neuronal morphology was analysed from 8 to 14 neurons (Bregma 1.94 to −2.46 mm) per mouse (*n* = 2–4 mice/genotype). The number of spines was counted in 4 segments of primary and secondary dendrites per brain hemisphere.

### Statistical analysis

Sample sizes were determined according to own and other´s published results of comparable studies [38, 40, 41, 46, 48, 53]. During all experiments animals´ identities were numerically encoded, allowing the experimenter to be blinded to the genotype or experimental condition of each subject.

**Fig. 1:**
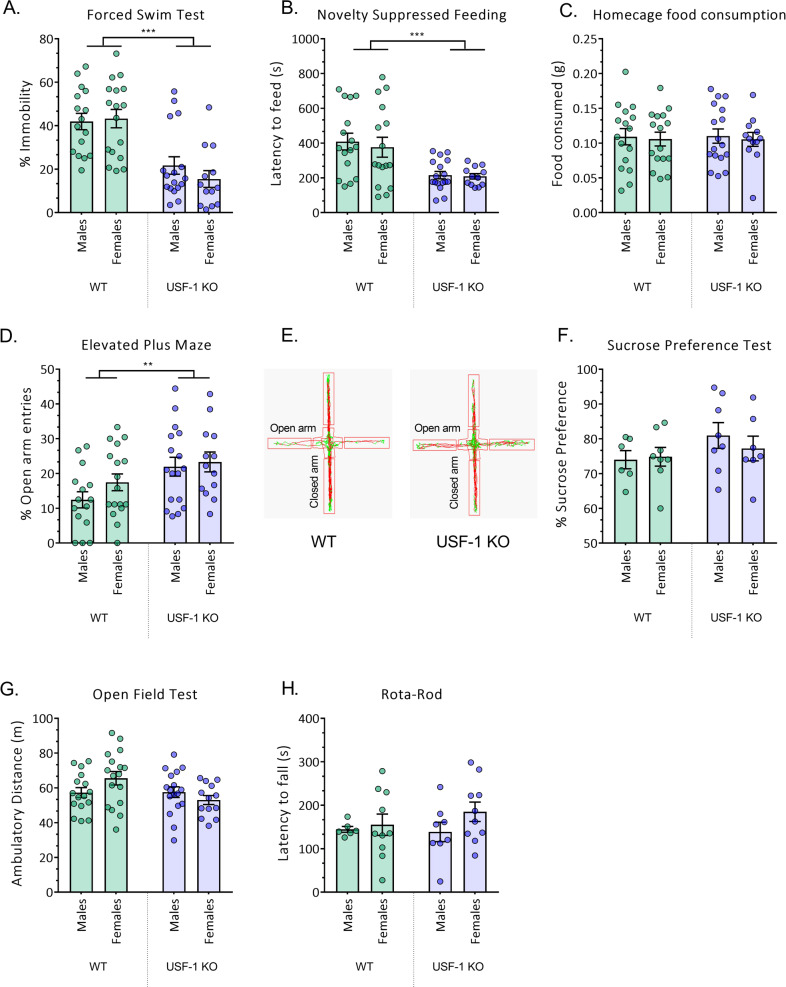
USF-1 deficiency ameliorates depression-like and anxiety-like behaviours. **A** Percentage (%) of immobility in the Forced Swim Test. **B** Latency to feed (s) in theNovelty Suppressed Feeding (NSF) test. **C** Post-NSF home-cage food consumption (g). **D** Percentage (%) of openarm entries in the Elevated Plus Maze (EPM). **E** Representative tracking images from the EPM. **F** Percentage (%) of sucrose preference **G** Ambulatory distance travelled in the Open Field (m). **H** Latency to fall (s) in the Rota Rod. All data are presented as mean ± SEM, *n* = 6–17 mice/group. Data are analysed by two-way ANOVA (genotype × sex). Only significant main effects of genotype are displayed; **p < 0.01, ***p < 0.001.

**Fig. 2:**
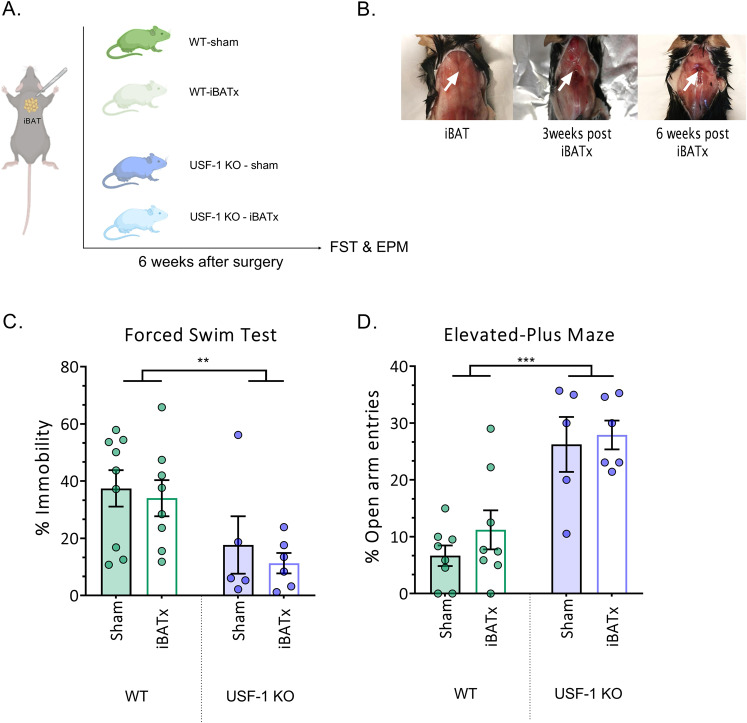
Surgical removal of interscapular BAT depots does not alter the behavioural phenotype of USF-1 KO mice. **A** Schematic illustration of the experimental procedure and timeline. WT and USF-1 KO mice were either subjected to surgical removal of interscapular BAT (iBATx) or to sham surgeries. The behavioural consequences of iBATx were evaluated 6 weeks after in the Forced Swim (FST) and Elevated Plus Maze (EPM) tests. **B** No sign of iBAT regeneration 6 weeks after surgical removal. **C** Percentage (%) of immobility in the FST. **D** Percentage (%) of open-arm entries in the EPM. Data are presented as mean ± SEM. Data were analysed by two-way ANOVA (genotype × iBATx). Only significant main effects of genotype are displayed. *n* = 5–9 female mice/group.; **p < 0.01, ***p < 0.001.

**Fig. 3:**
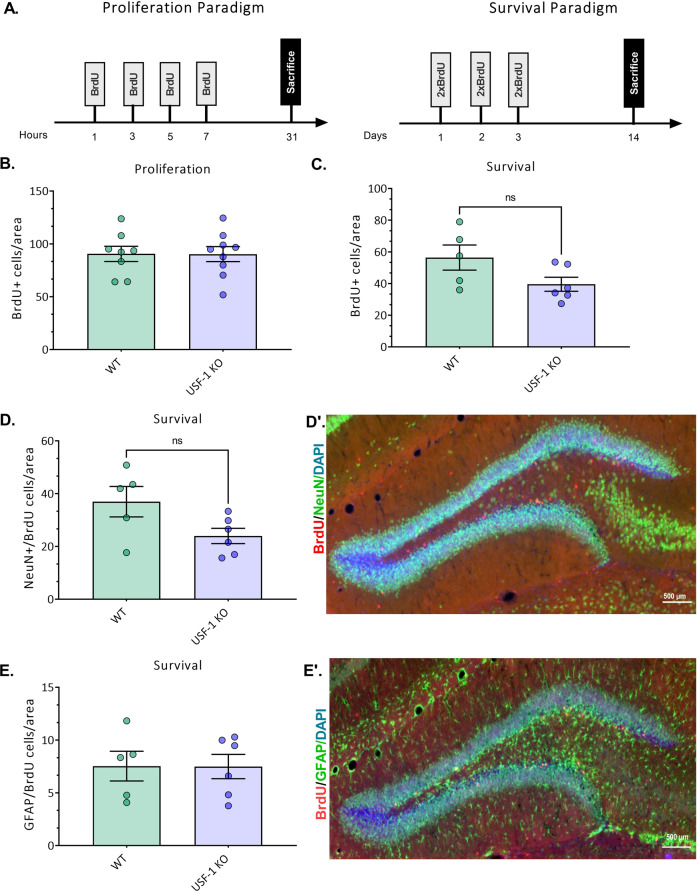
Adult hippocampal neurogenesis is not affected by USF-1 deficiency. **A** Diagrammatic representation of the experimental protocol. For the proliferation paradigm, mice were injected 4 times with BrdU, with a 2 h interval and euthanized 24 h after the last injection. For the survival paradigm, mice were injected twice daily, for 3 days and euthanized 14 days after the first injection. **B** Number of BrdU-positive cells in the proliferation paradigm, normalized to the size of the counted area. *n* = 8–9 mice/genotype (WT males:4; WT females: 4; USF-1 KO males:4; USF-1 KO females: 5). **C** Number of BrdU-positive cells in the survival paradigm, normalised to the size of the counted area. *n* = 5–6 mice/genotype (WT males:3; WT females: 2; USF-1 KO males:3; USF-1 KO females: 3). **D** Number of double-labelled BrdU and NeuN cells in the survival paradigm, normalised to the size of the counted area. *n* = 5–6 mice/genotype (WT males:3; WT females: 2; USF-1 KO males:3; USF-1 KO females: 3). **D’** Representative microscopy image of a WT hippocampus; Red: BrdU; Green: NeuN; Blue: Dapi; scalebar: 500 µm. **E** Number of double-labelled BrdU and GFAP cells in the survival paradigm, normalised to the size of the counted area. *n* = 5–6 mice/genotype (WT males: 3; WT females: 2; USF-1 KO males:3; USF-1 KO females: 3). **E’** Representative microscopy image of a WT hippocampus; Red: BrdU; Green: GFAP; Blue: Dapi; scalebar: 500 µm. Data are presented as mean ± SEM.

**Fig. 4:**
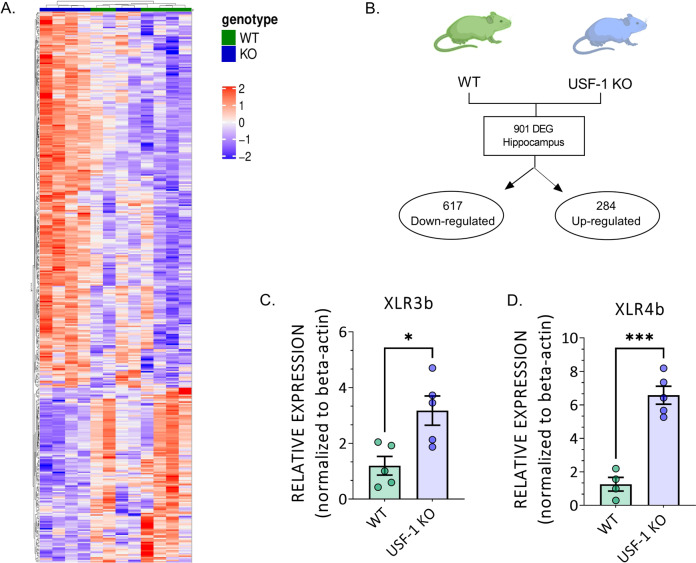
The transcriptomic profile of the USF-1 KO hippocampus presents with altered expression of Xlr genes. **A** Heatmap and **B** graphical illustration representing differentially expressed genes (DEG) in hippocampal tissue of USF-1 KO mice. **C** Relative hippocampal expression of xlr3b and **D** xlr4b in USF-1 KO and WT mice. *n* = 6 mice/genotype (WT males:3; WT females: 3; USF-1 KO males:3; USF-1 KO females: 3). Data are presented as mean ± SEM. Statistical significances resulting from student’s *t*-test are displayed; *p < 0.05, **p < 0.01, ***p < 0.001, n.s. not significant.

**Fig. 5:**
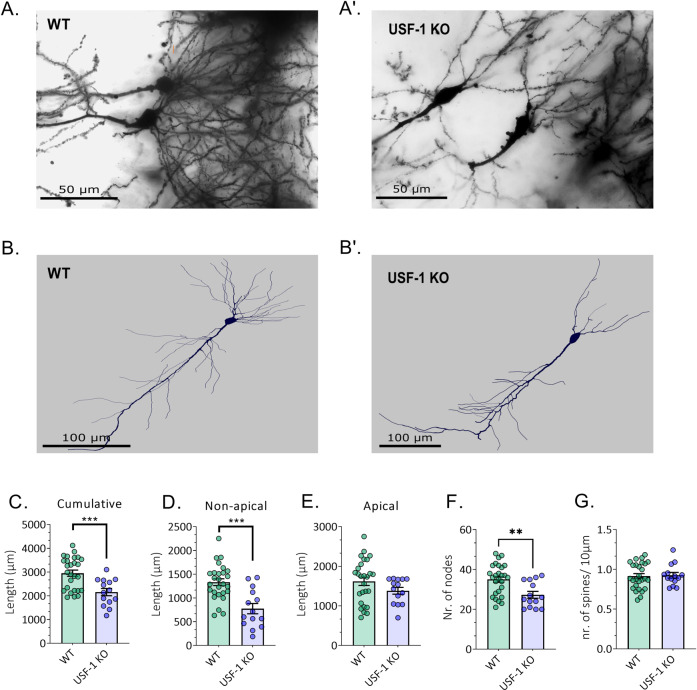
USF-1 deficiency leads to stubbed dendritic length and complexity. Examples of Golgi-Cox stained hippocampal brain sections of **A** WT and **A’** USF-1 KO mice (60× magnification; scalebar: 50 µm). Neurolucida reconstruction of pyramidal neurons in the hippocampus of **B** WT and **B’** USF-1 KO (scalebar: 100 µm). **C** Cumulative dendritic length (µm). **D** Length of non-apical dendrites (µm). **E** Length of apical dendrites (µm). **F** Number of nodes. **G** Spine density (nr. of spines/10 µm). Data are presented as mean ± SEM. *n* = 14–27 sections/ group. Statistical significances resulting from student’s *t*-test are displayed; **p < 0.01, ***p < 0.001.

GraphPad Prism 7.0 (GraphPad Software, San Diego, CA, USA) was used for all statistical analyses and preparation of graphs. Data were tested for normality using the Kolmogorov–Smirnov test prior to further statistical evaluation. The Tukey’s box plot method for the identification of statistical outliers was applied. Two-way analysis of variance (two-WAY ANOVA) was employed for the data displayed in Figs. 1 and 2. Student’s *t*-test was used for the analysis of data represented in Figs. 3, 4, 5 and Supplementary Fig. 1.

## Results

### USF-1 deficiency ameliorates depression-like and anxiety-like behaviours

In agreement with the original publication [37] we found that adult female and male USF-1 KO have reduced body weight compared to their WT littermates, while food intake normalized to body weight was unaltered (Supplementary Fig. 1A, B). These results confirm the earlier reports and corroborate the enhanced metabolic activity in this model.

Also, in line with the published data we did not observe genotype-dependent differences in the relative distribution of the BAT mass, inguinal white adipose tissue (iWAT) and epididymal white adipose tissue (eWAT) in either female or male animals (Suppl. Fig. 1C–E). We then used adult female and male USF-1 KO and WT controls for a comprehensive behavioural characterisation in a battery of standard paradigms, with special emphasis on tests assessing emotional behaviours relevant to depression and anxiety. In the FST where immobility reflects behavioural despair in response to exposure to an acute inescapable stress situation, USF-1 KO mice of both sexes were less immobile than their WT littermates (Fig. 1A; main effect of genotype *F* (1,59) = 35.46; *p* < 0.0001; *n* = 13–17/group). Further, in the NSF, in which animals are faced with the stressful conflict of pursuing food under anxiogenic conditions, both female and male USF-1 KO mice presented with shorter latencies to begin eating in the centre of a brightly illuminated novel arena than their WT counterparts. This suggests that USF-1-deficient animals are more prone to resolve the ambivalent situation in favour of the active behaviour to feed, despite a fear-inducing environment (Fig. 1B; main effect of genotype *F* (1,57) = 18.46; *p* < 0.0001 *n* = 12–17/group). However, food consumption in the home cage measured immediately after the NSF did not reveal any difference between genotype in either sex, confirming that the phenotype of USF-1 KO mice in the NSF was not due to a baseline alteration in the drive to eat (Fig. 1C). Notably, the displays in the FST and the NSF are considered as distinctive depression-related behavioural dimensions [54].

We also found the genotype-specific differences in depression-related behaviour in the FST and NSF to persist after chronic corticosterone treatment, considered a rodent model of depression [42, 43] (Supplementary Fig. 2A, B).

Exposure to the EPM allows for the determination of anxiety-like behaviour under fear-inducing conditions [55, 56]. Here, a significant main effect of genotype was noted with female and male USF-1-deficient mice displaying less anxiety face to the more aversive illuminated arms than WT littermates (Fig. 1D, E; *F* (1,58) = 8.734; *p* = 0.0045; *n* = 13–17/group).

Interestingly, in the SPT, which probes hedonic behaviour when animals are left undisturbed in their home cage, female and male USF-1 KO mice were undistinguishable from WT controls, suggesting no effect of USF-1 deficiency under baseline conditions in the absence of an external stressor (Fig. 1F).

USF-1 KO mice of both sexes travelled similar total distances as their WT littermates in the OFT evidencing no differences in exploratory and locomotor activity between genotypes (Fig. 1G). Similarly, motor coordination was unaltered in female and male KO mice as determined by the latency to fall in the RR (Fig. 1H), jointly dismissing unspecific biases through general behavioural alterations on the performance of USF-1 KO mice in the depression-related and anxiety-related tests.

#### The surgical removal of interscapular BAT depots (iBATX) does not alter the behavioural phenotype of USF-1-deficient mice

In order to investigate whether the reduction in stress-related-negative valence behaviours directly resulted from the highly active BAT of USF-1 KO mice, interscapular BAT, the largest BAT depot in rodents [57] corresponding to supraclavicular BAT in adult humans [58, 59] was surgically removed in female USF-1 KO and WT mice (Fig. 2A, B). 6 weeks post iBATx or sham surgery all animals were tested in the FST and the EPM. No effect of iBATx was found in either genotype. Instead, the previously observed phenotype of USF-1 KO mice was confirmed, with reduced immobility in the FST (Fig. 2C; *F* (1,24) = 9.53; *p* = 0.<01; *n* = 5–9/group) and a higher percentage of entries into the open arms in the EPM (Fig. 2D; *F* (1,23) = 32.64; *p* < 0.0001; *n* = 5–9/group) in USF-1 KO mice. These results indicate that the behavioural phenotype resulting from USF-1 deficiency does not require the presence of iBAT in adult animals.

#### Adult hippocampal neurogenesis is not affected by USF-1 deficiency

Progenitor cell proliferation in the subgranular zone of the adult hippocampal dentate gyrus and survival of newly generated neurons has been strongly related to depression-like behaviour in different animal models [60]. We therefore sought to examine whether the reduction of depression-like and anxiety-like behaviour in USF-1 KO mice was also reflected in an alteration of adult hippocampal neurogenesis. We used BrDU-dependent immunofluorescence histochemistry and evaluated proliferation of progenitor cells and differentiation and survival of newly born cells in the dentate gyrus (Fig. 3A). The number of BrDU+ cells was comparable between USF-1 KO and WT littermate controls both 24 h (Fig. 3B) and 14 days after BrDU administration (Fig. 3C). Similarly, the rate of differentiation of newly born cells into neurons (as indicated by the presence of NeuN; Fig. 3D and representative image 3D’) or astrocytes (as indicated by GFAP staining; Fig. 3E and representative image 3E’) was not significantly different between genotypes, suggesting that the lack of USF-1 did not impact on the processes of adult hippocampal neurogenesis, and that neurogenic effects may likely not account for the behavioural phenotype of USF-1 KO mice.

#### Several members of the xlr gene family are upregulated in the USF-1 KO hippocampus

Considering that USF-1 is a ubiquitously expressed transcription factor and given that the behavioural phenotype of USF-1 KO mice appeared independent of the signals acutely deriving from BAT activity, we next decided to employ a hypothesis-free approach to reveal the molecular signature in the brain, paralleling the behavioural repercussions of USF-1 deficiency. To this end, hippocampal transcriptomic profiles were generated by mRNA sequencing and compared between USF-1 KO and WT controls (Fig. 4A). 901 genes were found to be significantly differentially expressed (DEG) between genotypes in the hippocampus USF-1-deficient mice (Fig. 4B, full dataset in Supplementary Table 1).

A closer look at the list of DEGs revealed that the expression of several members of the X-linked lymphocyte-regulated (xlr) gene family was significantly upregulated in the USF-1 KO hippocampus: *xlr5c, xlr3a, xlr3b, xlr4b, xlr3c* and *xlr4a* (Supplementary Table 1). The xlr gene family comprises closely related genes, encoding similar proteins which are considered to be relevant to chromatin modification and have been recently discovered as important regulators of neuronal structure and function in the mouse brain [61, 62]. qRT-PCR confirmed the significant increase in the expression of *xlr3b* (Fig. 4C, *p* < 0.05, *n* = 5/group) and *xlr4b* (Fig. 4D, *p* < 0.0001, *n* = 4–5/group), which had been specifically identified as regulators of dendritic complexity, spine number and morphology [61].

Additional bioinformatic analysis of the top 100 DEGs confirms most of the affected genes to be direct targets of USF-1 mediated transcription (Supplementary Fig. 3A) and suggests alterations in biological functions and molecular processes with relevance to dendritic outgrowth and complexity (Supplementary Fig. 3B, C).

#### USF-1 deficiency leads to stubbed dendritic length and complexity

We then went on to explore whether enhanced levels of xrl transcripts also related to alterations in neuronal structure in the USF-1 KO brain. To this end, we investigated neuronal morphology in in USF-1 KO and WT mice using single-cell reconstructions of Golgi-Cox stained hippocampal sections (Fig. 5A, B).

We focussed on pyramidal cells in the CA1, as previously the effects of xlr genes on neuronal structure in the cortex had also been examined in pyramidal cells [61]. We found a significant decrease in the cumulative dendritic length (Fig. 5C, *p* < 0.001, *n* = 14–27/group), specifically resulting from a highly significant reduction in the length of non-apical dendrites (Fig. 5D, *p* < 0.0001, *n* = 14–27/group), in line with previous observations related to increased expression of xlr3b and xlr4b [61]. Next, we asked whether USF-1 deficiency also impacted on dendritic arborisation and spine density. In comparison with WT controls, USF-1 KO neurons had a significantly lesser number of nodes (Fig. 5F, *p* < 0.01, *n* = 14–27/group), whereas no difference in spine density was observed (Fig. 5G). These observations demonstrate that lack of USF-1, concomitantly to the dysregulation of xlr gene expression, alters neuronal morphology in the mouse brain.

## Discussion

We here used a USF-1-deficient mouse strain as a genetic model to probe the consequences of constitutively activated BAT on affective behaviour, and to test the role of BAT activation as a mechanistic interrelator between metabolic and mood states. Combining behavioural, surgical and molecular tools, we demonstrate that absence of USF-1 positively modulates affective behaviour alongside its previously described beneficial impact on energy expenditure and lipid metabolism [37].

We postulated that, in analogy to the favourable shift in metabolic activity, the increase in BAT activity resulting from the lack of USF-1 may also lead to a reduction in the displays of negative affect in mice. Confirming our expectation, we found a significant reduction of depression-like and anxiety-like behaviours in USF-1 KO mice of both sexes. The performance of USF-1 KO mice differed from WT controls specifically under mildly stressful or anxiogenic conditions, suggesting the promotion of active coping strategies under trigger conditions and a modification of the corresponding phenotypic states, rather than a constitutive alteration of behavioural traits to result from USF-1 deficiency.

Considering our initial hypothesis, we went on to experimentally address whether the behavioural phenotype of USF-1 mice was a direct consequence of the highly active BAT, a hallmark of the USF-1-deficient mouse strain [37]. However, surgical removal of interscapular BAT did not affect the performance of either USF-1 KO or WT mice in the FST and EPM the two tests in which we had observed a salient USF-1-dependent behavioural phenotype. While these results strongly indicate that the behavioural phenotype of USF-1 KO mice is independent of their overactive BAT and may likely not result from direct BAT-to-brain effects, two major points have to be taken into consideration when interpreting these data: (i) Although no studies have yet specifically evaluated the ontogenetic profile of BAT activity in USF-1 KO mice, it is likely that the constitutive nature of the genotype may have also resulted in augmented BAT recruitment during development. Hence, we cannot exclude the possibility that increased BAT activity during earlier stages of development might have (directly or indirectly) induced persistent alterations contributing to the observed phenotype.

(ii) While iBAT is the largest BAT depot in rodents, other areas with BAT pads exist in mice and these regions were left intact during iBATx [63] and activity of these BAT depots may have contributed to behavioural alterations in USF-1 KO mice. It has to be noted that a recent study reports an increase in depression-like behaviour in WT mice subjected to iBATx [64], while we did not observe any behavioural of effects iBATx in either USF-1 KO or WT mice. This discrepancy may result from differences in the experimental design (behavioural testing 4 weeks or 6 weeks after iBATx), the sex of the animals, or housing conditions (group versus single housing).

In light of the strong experimental evidence linking adult hippocampal neurogenesis with depression and the effects of antidepressant treatments, we challenged USF-1 KO and WT mice with BrDU. However, as no differences between genotypes were observed for neural progenitor cell proliferation, differentiation, or the survival of newborn cells in the hippocampal dentate gyrus, we propose neurogenesis-independent mechanisms to underlie the behavioural phenotype of USF-1 KO mice.

Given that USF-1 is a ubiquitously expressed transcription factor likely directly and indirectly regulating the expression of a multitude of genes, we decided to employ an unbiased transcriptomic approach to shed light on the molecular processes accompanying USF-1 deficiency in the brain. RNA-Seq revealed the significant upregulation of several members of the xlr family of chromatin remodelling genes, the mouse orthologs of FAM9 gene family [61, 65], including xlr3b and xlr4b with specific functions in the regulation of neuronal structure and function [61, 62]. Indeed, we found that the increase in xlr3b and xlr4b expression in USF-1 KO mice was paralleled by modifications in the dendritic complexity. These observations are noteworthy as previously xlr3b and xlr4b over-expression was shown to impact the on the structure of cortical neurons [61], proposing xlr genes as downstream effectors of USF-1 in the brain. Related to the behavioural phenotype of US-F1 KO mice it is striking that in a mouse model of early life stress, a precipitating factor for the development of depression, reduced xlr4 levels in the brain of wildtype inbred mice of the C57Bl/6 strain was observed [62]. Together with our results, this suggests a bidirectional modulation of xlr4 gene expression in the context of depression-like behaviour, which warrants further targeted investigations.

No sex-dependent effects of USF-1 deletion were observed in the initial behavioural screen. In consideration of this aspect and against the background of the “3R policy” subsequently either males, females or mixed cohorts were used in order to reduce the number of experimental animals. However, given that a similar behavioural phenotype can have differing underlying molecular underpinnings and vice versa, this sex-specific mechanistic aspect remains unanswered and consitutes a limitation of the present study.

In summary, the findings of the present study identify USF-1 as a positive regulator of cardiometabolic health and behavioural functions related to active stress coping and depression-like behaviour, with a direct impact on the molecular and structural architecture of the brain. These data are also relevant in a translational framework, proposing that metabolic and mental disorders may, at least in part, involve common molecular mediators.

## Supplementary information

Suppl Figure 1

Suppl Figure 2

Suppl Figure 3

Suppl Figure Legends

Suppl Table 1
