# Supplementary figures and images for: The metabolic regulator USF-1 is involved in the control of affective behaviour in mice
Source: Transl Psychiatry. 2022 Dec 1;12:497. doi: 10.1038/s41398-022-02266-5 (PMC9712601; doi:10.1038/s41398-022-02266-5)

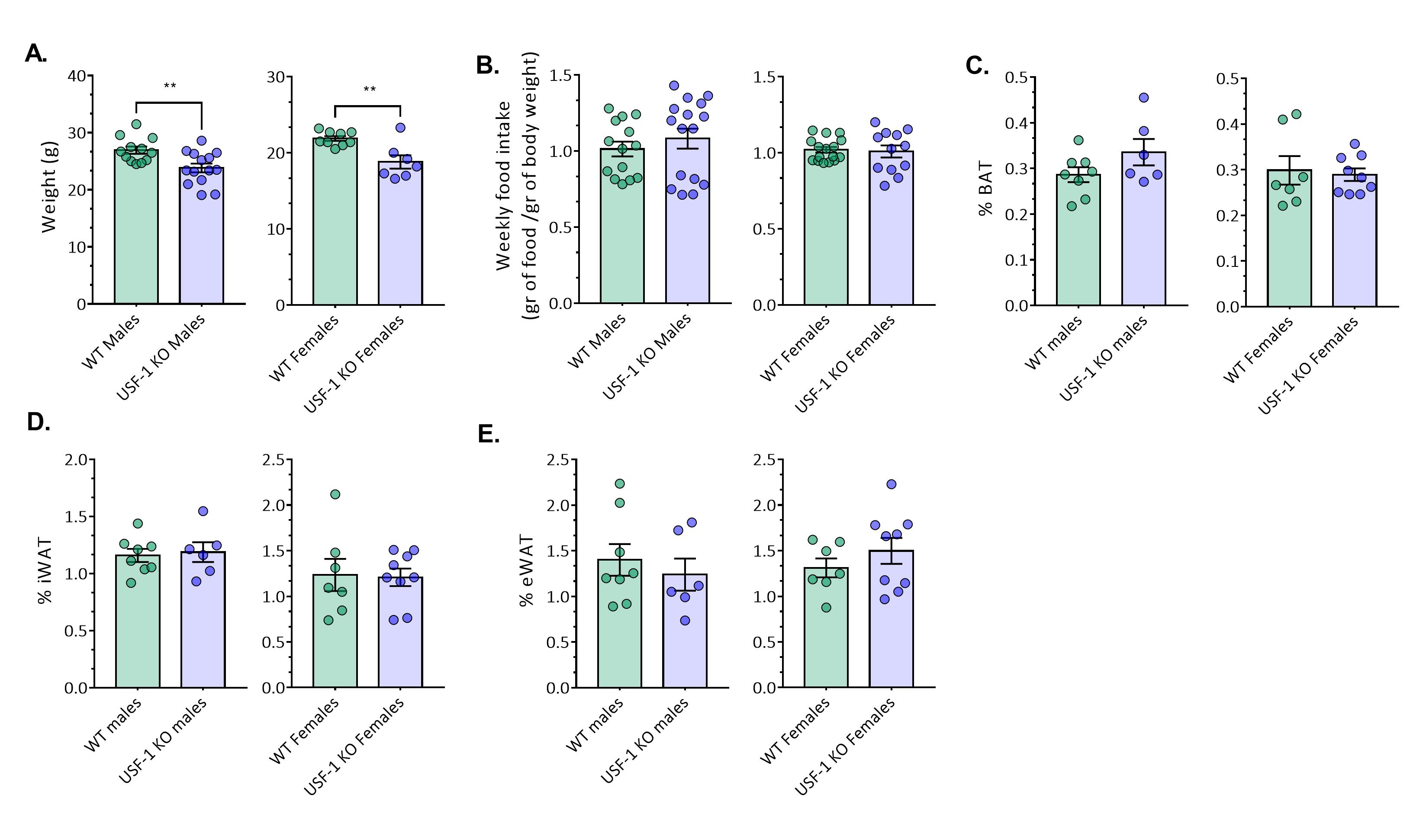

Supplement: Supplementary file 1 — Suppl Figure 1 [file 41398_2022_2266_MOESM1_ESM.jpg]

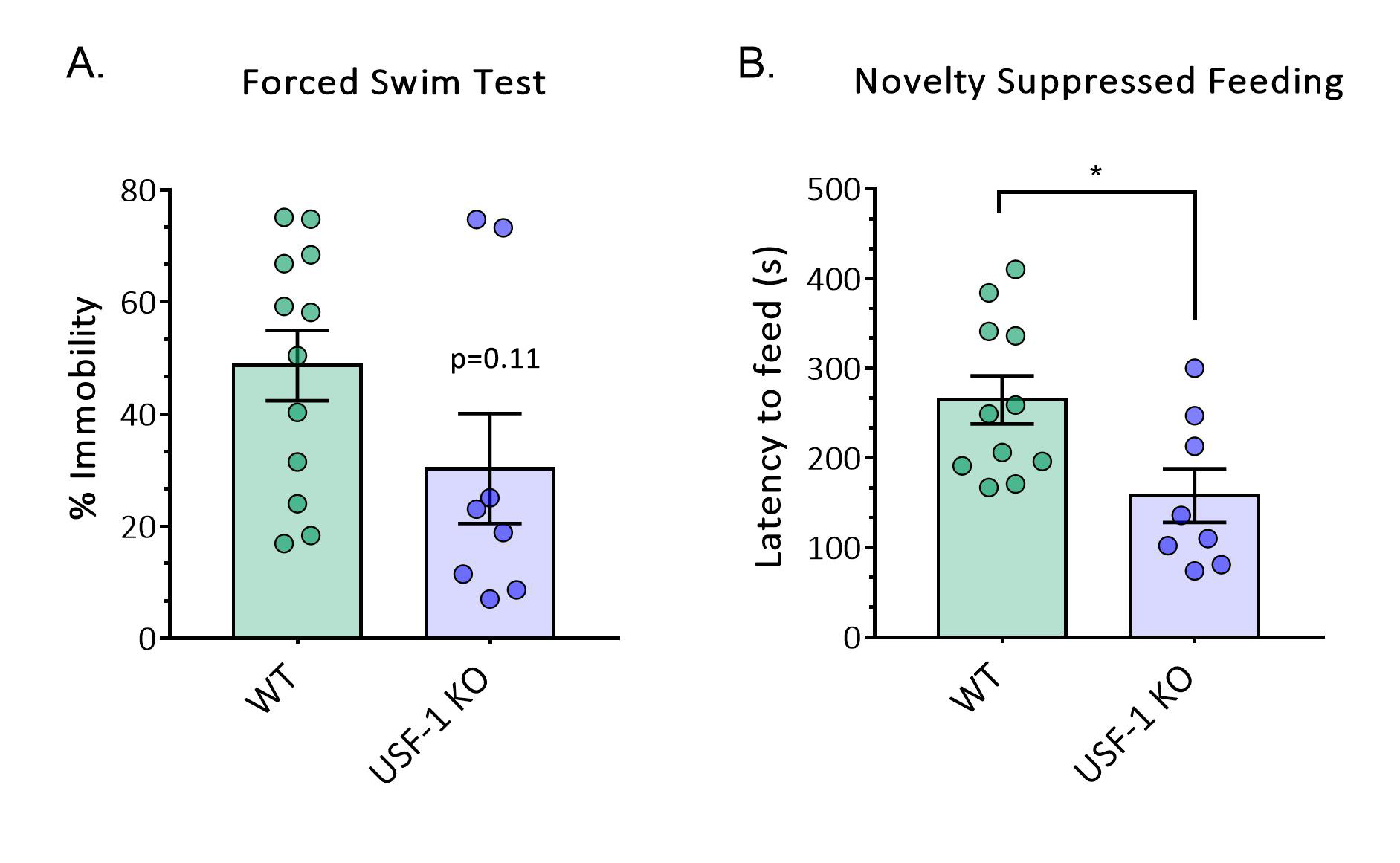

Supplement: Supplementary file 2 — Suppl Figure 2 [file 41398_2022_2266_MOESM2_ESM.jpg]

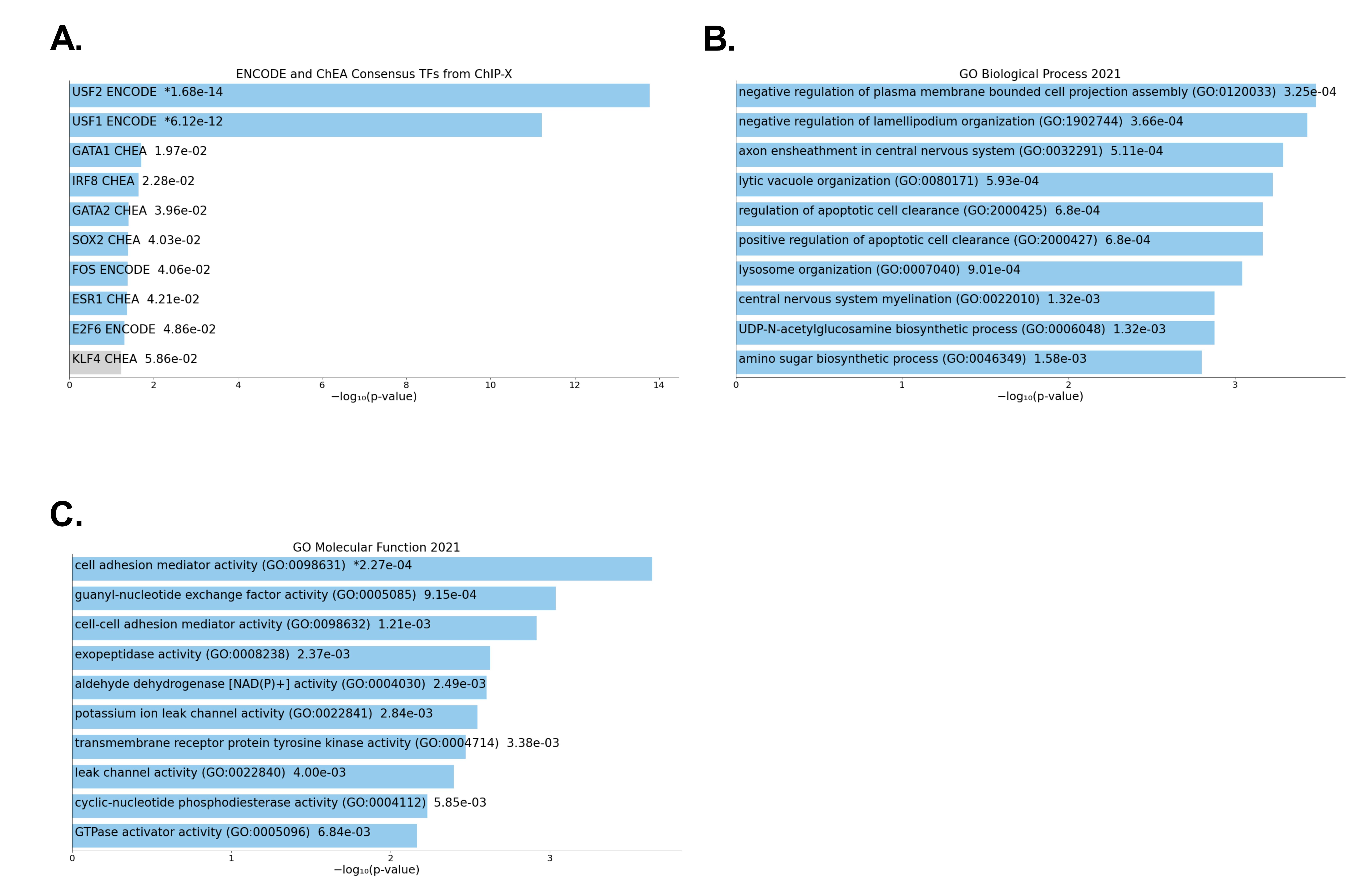

Supplement: Supplementary file 3 — Suppl Figure 3 [file 41398_2022_2266_MOESM3_ESM.jpg]
