# Supplementary material for: The metabolic regulator USF-1 is involved in the control of affective behaviour in mice
Source: Transl Psychiatry. 2022 Dec 1;12:497. doi: 10.1038/s41398-022-02266-5 (PMC9712601; doi:10.1038/s41398-022-02266-5)
Supplement: Supplementary file 4 — Suppl Figure Legends [file 41398_2022_2266_MOESM4_ESM.docx]

**Supplementary Figure 1. Body weight, food intake and fat composition of USF1 KO and WT mice.**

**A)** Body weight (g), **B)** weekly food intake normalized to body weight (grams of food/grams of body weight) **C)** relative interscapular BAT (iBAT) mass, **D)** relative inguinal WAT (iWAT) mass, **E)** relative epididymal WAT (eWAT) mass of WT and USF-1 KO mice, n=6-14 mice/ group. Data analysed with student’s t-test. Data are presented as mean ± SEM.

**Supplementary Figure 2. Behavioral effects of chronic corticosterone (CORT) treatment.**

**A)** Percentage (%) of immobility in the Forced Swim Test (FST). **B)** Latency to feed (s) in the Novelty Suppressed Feeding (NSF) test. Behavioral experiments were performed two weeks after the termination of the CORT exposure. All data are presented as mean ± SEM. Statistical significances resulting from student’s t-test are displayed; * p < 0.05. n= 8-12 mice/genotype (WT males:7; WT females: 5; USF-1 KO males:2; USF-1 KO females: 6). In NSF an outlier was detected and removed form the dataset (WT male).

**Supplementary Figure 3. Bioinformatic analysis of top 100 differentially expressed genes (DEG) in the USF1 KO hippocampus.**

**A)** ‘ENCODE and ChEA Consensus TFs from ChIP-X’ enrichment analysis of top 100 DEG. **B)** ‘GO Biological Process’ enrichment analysis of top 100 DEG. **C)** ‘GO Molecular Function’ enrichment analysis of top 100 DEG.
