# Supplementary material for: The metabolic regulator USF-1 is involved in the control of affective behaviour in mice
Source: Transl Psychiatry. 2022 Dec 1;12:497. doi: 10.1038/s41398-022-02266-5 (PMC9712601; doi:10.1038/s41398-022-02266-5)
Supplement: Supplementary file 5 — Suppl Table 1 [file 41398_2022_2266_MOESM5_ESM.pdf]

gene_name	gene_biotype	baseMean	log2FoldChange	pvalue	padj
Xlr5c	protein_coding	3.655661884	1.9044268	9.96E-06	0.02028416
Xlr3a	protein_coding	39.13325865	1.541007838	3.39E-07	2.31E-04
Tmem202	protein_coding	47.87208289	1.540354551	1.54E-13	5.52E-10
Xlr3b	protein_coding	110.8499599	1.160196112	3.03E-07	1.03E-04
Xlr4b	protein_coding	32.1669824	0.881552809	1.36E-05	0.004009814
Xlr3c	protein_coding	56.08310268	0.879928304	2.87E-06	0.001216685
Strc	protein_coding	93.91015178	0.876919442	8.39E-15	1.94E-11
Pgam2	protein_coding	201.6368071	0.731681968	5.46E-12	6.65E-09
Igsf9	protein_coding	145.3232775	0.673817512	9.15E-12	1.25E-08
C4b	protein_coding	1329.108458	0.54968276	2.81E-12	2.64E-09
3110082J24Rik	protein_coding	20.59478838	0.519072496	6.31E-05	0.018678272
Gm20033	processed_transcript	85.25740598	0.519028412	1.30E-05	0.001750051
C4a	processed_transcript	330.9752386	0.435506004	3.36E-09	1.89E-06
Gm29666	protein_coding	130.2888189	0.415009407	4.23E-06	8.52E-04
Uhmk1	protein_coding	9015.352399	0.3752966	1.29E-05	9.33E-04
Kcnk10	protein_coding	351.8540022	0.351159399	2.57E-07	6.52E-05
Htra4	protein_coding	78.34361315	0.335643463	4.51E-04	0.022160155
E330034L11Rik	TEC	203.2773233	0.330285192	2.13E-06	8.25E-04
Gm45552	antisense	42.93617583	0.327474706	4.66E-04	0.044734648
1700087I21Rik	lincRNA	328.1237696	0.321042178	5.49E-05	0.005626474
Ap1g2	protein_coding	302.0669115	0.318068119	1.45E-04	0.008160217
Stpg1	protein_coding	148.163323	0.314525925	2.19E-04	0.014654699
Fat4	protein_coding	1123.385405	0.308800799	2.25E-05	0.002794475
Zmym6	protein_coding	1058.031392	0.308091846	6.02E-09	2.03E-06
Olfr539	protein_coding	30.79644635	0.307155657	5.77E-04	0.0517859
Vangl2	protein_coding	413.7437342	0.304462301	2.92E-05	0.00364464
Gipc3	protein_coding	65.48801744	0.303102421	6.63E-04	0.031390408
Gm26703	antisense	71.21558145	0.289260983	6.91E-04	0.034571024
Stag3	protein_coding	50.41482313	0.281845667	7.63E-04	0.064663529
Dbp	protein_coding	1034.00388	0.280919496	1.96E-05	0.001623746
Xlr4a	protein_coding	15.65508236	0.279489118	1.58E-04	0.033904643
Efcab15	protein_coding	52.23582008	0.271300523	9.27E-04	0.071694349
9330121K16Rik	TEC	717.6116497	0.26375165	1.61E-04	0.008785194
Pcdh8	protein_coding	1350.184269	0.255312654	3.97E-04	0.019083237
Npy1r	protein_coding	766.9449965	0.254884137	5.12E-06	0.001191688
Jun	protein_coding	2139.839978	0.254586223	1.69E-05	0.001487111
Tiam1	protein_coding	3334.258313	0.253921438	1.30E-05	8.87E-04
Cd46	protein_coding	260.7968292	0.252633739	2.38E-05	0.002698266
Ltbp4	protein_coding	2045.669789	0.243830322	1.35E-05	9.39E-04
Gm48932	TEC	105.7411604	0.237931464	0.001064418	0.038901322
Gm37206	TEC	237.6740175	0.22804758	4.60E-04	0.021047823
Thap3	protein_coding	359.9649	0.226487028	0.000188068	0.012941899
Grm2	protein_coding	1678.726158	0.214589496	5.75E-04	0.014567343
Plod2	protein_coding	216.6778321	0.21296438	8.28E-04	0.026467401
Cebpd	protein_coding	188.9709234	0.205546355	0.00197321	0.066207193
Atxn7l2	protein_coding	753.6525938	0.199120964	1.07E-04	0.009220843
Uvssa	protein_coding	409.5170204	0.1932187	6.94E-04	0.032256828
Cdh23	protein_coding	81.63620231	0.191267286	0.002372389	0.079878427
Chadl	protein_coding	227.6311497	0.186025679	2.73E-04	0.0149275

Pcdhga12	protein_coding	374.0975515	0.184951349	0.001487815	0.054846462
Ciart	protein_coding	212.0561424	0.183424981	0.002328812	0.072204493
Cygb	protein_coding	1913.880836	0.180045767	2.03E-05	0.001216685
Lmtk3	protein_coding	9555.751023	0.178219629	1.65E-04	0.00785131
Pcdhgb7	protein_coding	167.96712	0.17664911	0.002250914	0.07055518
4933439C10Rik	processed_transcript	303.8611401	0.176385101	2.22E-04	0.012941899
Glis3	protein_coding	469.7476152	0.174313186	0.002587063	0.058387545
3110053B16Rik	transcribed_unprocessed_p	69.02797029	0.173175011	0.002678339	0.079424364
Prr36	protein_coding	1402.149539	0.169539673	2.06E-04	0.009206716
Dffb	protein_coding	199.3033741	0.167748839	5.39E-04	0.026537018
Gm47694	TEC	261.4297601	0.162938847	0.001600632	0.056869455
Muc3a	protein_coding	556.1585515	0.161593521	0.00187143	0.075406157
1810010H24Rik	protein_coding	83.63173659	0.161231332	0.00371552	0.096668811
Pxylp1	protein_coding	370.8236123	0.161031192	7.22E-04	0.032256828
Rreb1	protein_coding	2704.140489	0.160741102	0.002365006	0.036421798
Gm35339	protein_coding	440.536658	0.159760653	0.001099887	0.044032875
Gm15328	lincRNA	150.1427213	0.159610494	0.002245701	0.088193226
Bmp1	protein_coding	2503.015946	0.159316848	0.001745713	0.038632639
Zkscan2	protein_coding	1077.743578	0.158960365	7.01E-04	0.021629863
Aste1	protein_coding	118.742642	0.155183167	0.00201676	0.098562143
Cxcl12	protein_coding	1167.642033	0.154739574	8.29E-04	0.033904643
Pkia	protein_coding	3791.114269	0.154049202	1.80E-06	1.52E-04
Lrfn3	protein_coding	636.99367	0.153578773	6.75E-05	0.007915869
Prr12	protein_coding	2756.547089	0.151846535	6.99E-05	0.003454201
Mctp1	protein_coding	1250.784893	0.150793787	0.001831269	0.032218818
Tnrc6c	protein_coding	3824.469327	0.149514902	1.97E-04	0.005626474
Shc2	protein_coding	1250.772401	0.148898793	3.07E-04	0.014500399
Zfp46	protein_coding	1273.469645	0.148573825	0.003474147	0.073356728
Pnck	protein_coding	1695.33036	0.147321487	0.001736069	0.031110687
Npr2	protein_coding	1774.286303	0.145283329	1.45E-05	0.001782642
Zfp512b	protein_coding	3107.84919	0.14216118	6.32E-04	0.015580081
Srsf12	protein_coding	552.2337333	0.141863633	9.36E-05	0.010084286
Coro7	protein_coding	2253.210951	0.138306087	8.45E-04	0.018883964
Zc3h6	protein_coding	398.7248547	0.13654668	0.002138742	0.067110252
Tagln2	protein_coding	249.1200315	0.135996495	0.003738212	0.08639469
Igsf3	protein_coding	637.1263895	0.135059694	0.001831386	0.074279396
AI480526	lincRNA	1114.854789	0.134097714	4.09E-04	0.014567343
Susd1	protein_coding	283.1694528	0.133878923	0.003395944	0.091546496
Myt1	protein_coding	399.2137601	0.133707587	0.002074388	0.066266398
Spaca6	protein_coding	428.9352996	0.130296947	0.002967768	0.086990992
Pnmal2	protein_coding	13083.58527	0.129374003	1.63E-04	0.007828905
Zxdc	protein_coding	632.0467524	0.128472643	4.68E-04	0.018678272
Hnrnpa0	protein_coding	2103.398987	0.1279565	3.97E-04	0.014567343
Mfsd4a	protein_coding	1660.38771	0.127798302	0.004761557	0.089725795
Btg3	protein_coding	313.2572531	0.126807059	0.002432062	0.066247407
Celf3	protein_coding	5716.066078	0.126433053	7.09E-04	0.021718286
Cdc25b	protein_coding	763.1380577	0.126428491	0.001097683	0.053737528
Zfp865	protein_coding	679.8980565	0.126404089	8.29E-04	0.026467401
Rbm3	protein_coding	1234.594888	0.123142199	0.003901732	0.068134745
Morc2a	protein_coding	1535.400506	0.123076296	5.53E-05	0.004961117

Prelid3a	protein_coding	591.7523864	0.122938414	0.002070393	0.080395691
Sdhc	protein_coding	1553.278735	0.120894868	0.001334674	0.03275826
Fam57b	protein_coding	1977.001037	0.120610429	4.12E-04	0.011354844
Nova2	protein_coding	3643.391854	0.118885463	7.56E-04	0.022160155
Cops7a	protein_coding	2922.79112	0.117736522	6.08E-05	0.004009814
Slc6a6	protein_coding	4032.484909	0.117575429	0.001255264	0.018883964
Adam1a	protein_coding	351.2653758	0.117478612	0.002973454	0.08438638
Ppt1	protein_coding	2341.395377	0.11587186	2.42E-04	0.010157754
Whamm	protein_coding	511.1290831	0.115341665	8.85E-04	0.036245621
Gm42829	sense_intronic	273.7614205	0.115294005	0.002561009	0.066385105
Foxo1	protein_coding	1097.66976	0.114896887	0.003202232	0.058387545
H1f10	protein_coding	459.1939607	0.114616103	0.001898727	0.062654578
Pxn	protein_coding	1661.451017	0.114255124	0.003779907	0.066385105
Krba1	protein_coding	1781.643708	0.114044284	4.53E-05	0.004308584
Sema5a	protein_coding	3253.785567	0.113928893	0.007772635	0.079568717
Dennd1a	protein_coding	2281.372979	0.113411018	9.89E-05	0.004271214
Fbrsl1	protein_coding	1727.48301	0.112881781	0.001675338	0.05055761
Sf3a2	protein_coding	1048.144019	0.112711897	7.98E-04	0.030466724
Fndc10	protein_coding	633.0153315	0.112481271	0.00276374	0.082514434
Usp42	protein_coding	701.7301144	0.112213026	0.001011	0.050457797
Cbx4	protein_coding	1265.390406	0.110647287	0.001511396	0.035027202
Syt7	protein_coding	12854.44143	0.110028396	0.002625082	0.030826846
Mtf2	protein_coding	1087.458892	0.108816261	4.68E-04	0.022874553
Miat	lincRNA	20233.04324	0.108766844	9.71E-04	0.015594939
Zfp777	protein_coding	855.8090569	0.108512976	0.001250753	0.031129698
Map1s	protein_coding	1859.658321	0.108440918	0.002107701	0.034571024
Pcif1	protein_coding	1523.572702	0.108359216	2.22E-04	0.012857381
Marcksl1	protein_coding	1715.830563	0.108044858	0.002932229	0.043489531
Mapk1ip1	protein_coding	1175.189005	0.107886416	1.59E-04	0.0076895
Dchs1	protein_coding	704.9741357	0.107217088	0.001395693	0.037809048
Zfp362	protein_coding	776.6544371	0.107097861	7.23E-04	0.039118281
Ets2	protein_coding	1893.708057	0.106659675	0.002210137	0.045699485
Fance	protein_coding	362.3821163	0.106434211	0.002349193	0.054846462
Rbm33	protein_coding	2982.001411	0.105467356	0.001889545	0.039970066
Fbxl20	protein_coding	1499.114263	0.103423355	5.98E-04	0.0149275
Grina	protein_coding	12849.48233	0.103370672	2.40E-04	0.006447002
Atp6v1a	protein_coding	13012.37291	0.102523143	3.08E-04	0.006982479
Mpg	protein_coding	284.2110417	0.102165153	0.004868139	0.099535834
Ache	protein_coding	1374.320341	0.101903731	7.55E-04	0.017834441
Tmem201	protein_coding	2432.424963	0.101340394	0.001373157	0.025944587
Tm7sf2	protein_coding	395.1589608	0.101325231	0.003521414	0.094946509
Ccsap	protein_coding	1300.5663	0.100876302	0.004520222	0.095405136
Plekhg5	protein_coding	5050.381219	0.100779437	0.007581358	0.081650322
Zswim8	protein_coding	5464.981377	0.100656357	0.00101962	0.02172015
Tafa2	protein_coding	1194.583313	0.099727717	0.004202264	0.070039363
Arhgef1	protein_coding	1923.569181	0.099666373	8.92E-04	0.024692898
Unc5a	protein_coding	4732.526398	0.099219405	0.003636682	0.050734306
Retreg2	protein_coding	4843.166353	0.098989908	2.00E-04	0.005318007
Sox12	protein_coding	806.8889446	0.098961887	0.003815005	0.093284171
Plekha5	protein_coding	2155.486286	0.098795746	4.27E-04	0.011642243

Carmil2	protein_coding	1702.55381	0.09875895	0.00266425	0.051668673
Jund	protein_coding	1600.612347	0.098423164	0.00453617	0.058948794
Slitrk3	protein_coding	2323.068947	0.098200928	0.006516161	0.091761093
Pde1b	protein_coding	3648.241492	0.096264787	0.001412755	0.018678272
Tbkbp1	protein_coding	2213.692244	0.096257459	8.05E-04	0.018316655
Rap2b	protein_coding	1527.278603	0.095560857	0.006117119	0.088372533
Prdm10	protein_coding	751.8113899	0.095555037	0.003093848	0.088745667
Lmo7	protein_coding	3444.368934	0.095474481	0.010513879	0.098738598
Tomm20	protein_coding	7828.551001	0.095014622	7.31E-04	0.021965581
Ptbp2	protein_coding	2281.651737	0.095013135	4.25E-04	0.011617749
Gm3764	lincRNA	2196.078639	0.094851706	0.003928292	0.053851645
Usp11	protein_coding	4583.915704	0.094572342	0.001080893	0.018678272
Pkd1	protein_coding	5691.428736	0.094467383	0.002835068	0.054846462
N4bp1	protein_coding	1760.474211	0.094414412	0.001429148	0.044723843
Fgd1	protein_coding	508.9356274	0.094369076	0.003882398	0.075928047
6330403L08Rik	antisense	811.9191638	0.094152403	0.003128514	0.077625181
St6galnac5	protein_coding	5759.176777	0.093426408	0.00664313	0.094657366
Unk	protein_coding	722.3717986	0.093328871	0.003057264	0.08822077
Ubald1	protein_coding	2978.616736	0.093124507	0.001779738	0.031567812
Fgf14	protein_coding	608.3924443	0.092747747	0.003071558	0.066247407
Nrg3	protein_coding	981.9386892	0.092361162	0.003822926	0.066266398
Dcaf7	protein_coding	6636.937819	0.092329055	0.001910675	0.022160155
Auts2	protein_coding	2759.344592	0.092094769	0.007734006	0.079424364
Wdr33	protein_coding	1671.703507	0.091930063	3.06E-04	0.014497385
Fto	protein_coding	4700.604492	0.091703639	8.81E-05	0.004009814
Nfrkb	protein_coding	1038.430491	0.090206352	0.003451186	0.080314409
Tmem179	protein_coding	3415.687044	0.090097354	0.002194818	0.045458125
Foxp4	protein_coding	1418.388334	0.089795773	0.002208584	0.061207841
Phyhip	protein_coding	17601.5644	0.089784263	0.004591131	0.059556513
Safb2	protein_coding	2588.100095	0.089716479	0.001104592	0.022160155
Gng2	protein_coding	7069.031563	0.08926343	0.005333818	0.044365705
Ufd1	protein_coding	1510.098008	0.089027694	7.82E-04	0.022195352
Fbrs	protein_coding	1651.773371	0.088872373	3.22E-04	0.012545809
Atg13	protein_coding	2351.47619	0.088572063	5.95E-04	0.01897351
C130071C03Rik	lincRNA	1535.786433	0.088547857	0.004795536	0.099535834
Slc45a1	protein_coding	1274.21081	0.088533277	0.001125633	0.037896168
Tspoap1	protein_coding	10091.48971	0.088192705	0.002942968	0.035349477
Flnb	protein_coding	3430.701307	0.088119893	0.004829736	0.07770181
Rbfox3	protein_coding	7766.85156	0.087867203	0.006762719	0.058243586
Ppp6r2	protein_coding	2616.727592	0.087720411	0.002444424	0.038142197
Rgmb	protein_coding	2152.259614	0.08758498	0.00476533	0.07716347
Tmem59l	protein_coding	7307.669558	0.087472368	0.004223738	0.045458125
Alg2	protein_coding	3509.884602	0.087308514	7.83E-04	0.01355988
Leng8	protein_coding	10934.25259	0.086825012	0.003445945	0.033277079
Tnik	protein_coding	5666.018824	0.086534853	0.003266963	0.032256828
Ptms	protein_coding	12389.25409	0.086301926	0.001142958	0.019083237
Med25	protein_coding	2368.271533	0.085830378	0.001347411	0.033005923
Pigt	protein_coding	2069.225513	0.085758049	0.001098903	0.022263147
Igfbp5	protein_coding	6508.848343	0.085742899	0.00758855	0.057329313
Bcl7a	protein_coding	2747.423134	0.085025791	0.003180761	0.058243586

Slit1	protein_coding	8312.803002	0.084829598	0.012904355	0.088154509
Med24	protein_coding	3613.649632	0.08472794	0.004082051	0.044416839
Zfand5	protein_coding	3167.597268	0.084079731	0.006185172	0.07215724
Grid1	protein_coding	1961.639858	0.084074759	0.003041354	0.04357186
Ilf3	protein_coding	2561.929666	0.083894359	6.74E-04	0.020583397
Kat6b	protein_coding	1706.286569	0.082350302	0.007434914	0.080653045
Atp6v0a1	protein_coding	15505.31795	0.081727657	0.003420815	0.03595324
Lhfpl4	protein_coding	3905.60143	0.081720695	6.61E-04	0.012039251
Rps6kl1	protein_coding	1444.098346	0.081475709	0.007036372	0.098134283
Ube2i	protein_coding	1326.671271	0.08143647	0.003483028	0.049545038
Zfp335	protein_coding	1332.388268	0.081430176	0.002532482	0.049993124
Sqstm1	protein_coding	10231.07688	0.081194997	6.33E-05	0.002219986
Safb	protein_coding	3917.00557	0.08111355	3.47E-04	0.013249421
Rasgrf1	protein_coding	18536.16449	0.081041794	0.009558819	0.066385105
Fbxo38	protein_coding	1317.20808	0.081010809	0.001874686	0.039864483
Vstm2a	protein_coding	2394.182661	0.080793229	0.004708022	0.057526838
Pdcd4	protein_coding	1881.977405	0.080534922	0.00665042	0.094657366
Cul9	protein_coding	2845.713308	0.080497493	0.007688116	0.080653045
Lsamp	protein_coding	10016.56284	0.080384193	0.004515797	0.039211889
Ywhaz	protein_coding	23428.05255	0.080380095	1.44E-04	0.003835067
Rgs11	protein_coding	1121.53412	0.080012083	0.004705492	0.097244712
Vps11	protein_coding	2053.932743	0.079995938	0.00360045	0.064082881
Itm2c	protein_coding	19336.5437	0.07980801	0.004376062	0.038565794
Keap1	protein_coding	1825.244614	0.079673506	0.001909384	0.040179362
Spin1	protein_coding	4080.769105	0.07914824	0.005755208	0.087084981
Gatad2b	protein_coding	2851.410686	0.078533419	0.005855081	0.070039363
1500011B03Rik	protein_coding	1899.211208	0.078510278	0.003130662	0.043117057
Ehmt2	protein_coding	5927.425046	0.078181071	0.006480294	0.056664738
Btbd3	protein_coding	6165.27283	0.078016457	0.015807826	0.091135377
Hmg20a	protein_coding	2612.217854	0.077923678	0.002872759	0.04297417
Tbpl1	protein_coding	1313.211782	0.077800546	0.003482844	0.080795317
Csnk1g3	protein_coding	1299.080116	0.077656217	0.005278733	0.081650322
Tmem170b	protein_coding	2110.85788	0.077633942	0.004420516	0.073098948
Elmod1	protein_coding	5746.547079	0.077468131	0.013653905	0.090827842
Sez6l	protein_coding	10289.72774	0.077151537	0.010827239	0.071998687
Amigo1	protein_coding	3043.912544	0.076817601	0.00211271	0.034160063
Wbp2	protein_coding	10059.18827	0.076806563	0.002402895	0.037809048
Otub1	protein_coding	5434.491979	0.076006429	0.00438675	0.038565794
Srebf2	protein_coding	6102.105313	0.075195784	0.003538793	0.036682738
Rab12	protein_coding	1906.902813	0.075122237	0.006340834	0.090525525
Atp13a1	protein_coding	1734.385393	0.074861262	0.004209796	0.070039363
Slc8a2	protein_coding	12190.48559	0.074358777	0.018201459	0.099810696
Prkaca	protein_coding	4534.530642	0.074349157	8.45E-04	0.019083237
Arhgap21	protein_coding	6951.826383	0.074335961	0.008815671	0.074999248
Hnrnpd	protein_coding	3568.687554	0.073967489	0.003162945	0.058948794
Srrm2	protein_coding	23139.58763	0.073897301	0.003742889	0.05180024
Ptprs	protein_coding	14289.37249	0.073765861	0.008353163	0.061233171
Nfix	protein_coding	7590.109706	0.073470362	0.008957128	0.070039363
Akap8l	protein_coding	3715.40626	0.073204965	0.004048868	0.04420436
Wbp11	protein_coding	2653.760543	0.072753465	0.006867682	0.094916762

Ksr2	protein_coding	2018.888656	0.072301977	0.010074018	0.098134283
Srrt	protein_coding	3576.834951	0.071637385	0.004481587	0.043489531
Tmem250-ps	protein_coding	1682.961101	0.071593517	0.0080831	0.085777226
Cpsf6	protein_coding	2821.55647	0.070881102	0.006414465	0.090827842
R3hdm4	protein_coding	6984.825662	0.070125466	0.012939303	0.080314409
Pithd1	protein_coding	1515.834025	0.070089817	0.008288507	0.087212892
Cry2	protein_coding	4151.153587	0.069245847	0.014544878	0.087106748
Bri3bp	protein_coding	3164.524613	0.068953721	0.007691936	0.082636437
Rtl6	protein_coding	2361.183031	0.067879044	0.009067185	0.090179012
Rab3a	protein_coding	12482.62298	0.067159068	0.005048466	0.047194071
Dgkg	protein_coding	3847.650434	0.066915564	0.017094563	0.095435596
Ssrp1	protein_coding	2700.954378	0.06683483	0.0056252	0.068377994
Klhdc3	protein_coding	4238.542573	0.066680838	0.007319181	0.080314409
Lrrc4b	protein_coding	3961.96626	0.065857003	0.009470162	0.06627479
Snrnp70	protein_coding	6703.019209	0.065548893	0.008983347	0.070039363
Gnl3l	protein_coding	4558.901422	0.065437149	0.009186055	0.07055518
Colgalt1	protein_coding	1501.770172	0.065316633	0.009465061	0.094382875
Ankrd11	protein_coding	6712.565377	0.065096816	0.012735292	0.087419864
Caly	protein_coding	4546.491093	0.065087082	0.012239517	0.091927023
Usp48	protein_coding	2974.021333	0.064686494	0.008891425	0.090684893
Brsk2	protein_coding	4608.374886	0.064654323	0.013178518	0.096081505
Necap1	protein_coding	4162.467428	0.064258413	0.010969143	0.08639469
Glyr1	protein_coding	5446.47097	0.064102733	0.00212295	0.026117369
Rtf1	protein_coding	2403.656503	0.063958202	0.010944965	0.098134283
Tpm1	protein_coding	4032.434366	0.063841359	0.011549836	0.074999248
Tmx4	protein_coding	5623.153024	0.063081007	0.007927373	0.058721769
Hnrnpul1	protein_coding	2996.517484	0.061860778	0.006008045	0.088935864
Edc4	protein_coding	2487.912569	0.061855125	0.010161479	0.098567408
Cds2	protein_coding	17650.62568	0.061144188	0.007891896	0.065196526
Apc2	protein_coding	5124.166339	0.060772564	0.016585176	0.094296589
Lonp1	protein_coding	2221.249071	0.060161975	0.007437517	0.077323139
Cpsf7	protein_coding	2299.137343	0.058561679	0.009303392	0.088745667
Gnb2	protein_coding	5879.527185	0.05813443	0.01228662	0.085371065
Atp6v0d1	protein_coding	4763.535404	0.057416121	0.008911896	0.075520165
Brd2	protein_coding	4781.780338	0.056709349	0.005514321	0.084361426
Folr1	protein_coding	164.0550348	-0.050886687	0.002757212	0.080314409
Pdha1	protein_coding	5337.55963	-0.0557768	0.008113474	0.059742029
4930402H24Rik	protein_coding	3647.378441	-0.05694001	0.010927196	0.072274285
Gnai1	protein_coding	3543.986849	-0.057824886	0.016734978	0.094603974
Ace	protein_coding	684.5136404	-0.06118445	0.002885057	0.085187605
Kcne2	protein_coding	110.0977064	-0.061357032	0.001613081	0.057122327
Xpo1	protein_coding	1935.871351	-0.065436237	0.005828363	0.066266398
Eps15	protein_coding	6638.825243	-0.066515978	0.01268811	0.094382875
Pdia3	protein_coding	4020.905868	-0.067760715	0.014966839	0.088243299
Acaca	protein_coding	2007.020623	-0.069026058	0.007722787	0.080815485
Sulf1	protein_coding	413.1368034	-0.069661525	0.001906835	0.066247407
Tex264	protein_coding	1340.109065	-0.069799847	0.006788564	0.095533058
Ktn1	protein_coding	2238.11042	-0.070721882	0.003405657	0.048523877
Rps12	protein_coding	2257.284729	-0.072691471	0.00888605	0.090684893
Akt2	protein_coding	2218.031513	-0.075308547	0.006760636	0.072447234

Picalm	protein_coding	3382.022074	-0.075617928	0.004291752	0.057122327
Usp30	protein_coding	1220.02793	-0.076037834	0.003676037	0.076206533
Uqcrh	protein_coding	2644.89581	-0.0763267	0.010011695	0.095405136
Cc2d1b	protein_coding	825.4424893	-0.077587235	0.005858477	0.087900868
Enoph1	protein_coding	1041.987487	-0.078402085	0.004004853	0.069256533
Lpgat1	protein_coding	4668.246036	-0.078997266	0.00666917	0.05230304
Neo1	protein_coding	2714.496486	-0.079760193	0.003698749	0.066247407
Fth1	protein_coding	41253.02576	-0.080182143	0.007800149	0.064682926
Pdk2	protein_coding	2918.037284	-0.081073812	0.003636766	0.049977135
Mettl16	protein_coding	841.0564687	-0.081932893	0.006323027	0.09037775
Ak3	protein_coding	1550.523266	-0.082233974	0.005115791	0.080314409
Mpp5	protein_coding	935.1218549	-0.082820686	0.004497816	0.072638065
Tbc1d9b	protein_coding	5495.67875	-0.084274977	0.005888935	0.057942475
Serf2	protein_coding	1559.259154	-0.08452237	0.001946067	0.050445378
Fermt2	protein_coding	1887.240476	-0.08532215	0.002099181	0.043489531
Hspd1	protein_coding	3129.315745	-0.085684057	3.76E-04	0.014176547
Hadha	protein_coding	1599.983522	-0.086381086	0.004757913	0.089725795
Hspe1	protein_coding	855.5270004	-0.08700976	0.002367316	0.069338434
Gde1	protein_coding	3506.536899	-0.087468292	1.30E-04	0.005301348
Acss1	protein_coding	859.3015042	-0.087963548	0.004066694	0.068747146
Pdgfa	protein_coding	719.262138	-0.088103676	0.002700536	0.095098113
Cacybp	protein_coding	1302.583181	-0.088613794	6.93E-04	0.021011978
Scd2	protein_coding	37688.7676	-0.088969765	0.004659081	0.075928047
Slc25a20	protein_coding	274.5682186	-0.089088337	0.005615146	0.095372544
Eef1b2	protein_coding	2017.512393	-0.089596604	0.001435615	0.025290793
Snx5	protein_coding	1601.952575	-0.089769266	0.001460142	0.04542821
Tulp4	protein_coding	2515.062728	-0.089769303	0.001049309	0.021718286
Wwp1	protein_coding	871.1351319	-0.090210549	0.00429547	0.091761093
Kcnj13	protein_coding	149.2326942	-0.090236985	9.26E-04	0.038303903
Chchd3	protein_coding	1035.171731	-0.090497797	0.001480621	0.034571024
Dst	protein_coding	12216.95926	-0.090815685	5.77E-04	0.010928194
Atp6v0a2	protein_coding	1798.463702	-0.090946344	0.00195456	0.050501287
Fnbp1	protein_coding	4939.126932	-0.091093851	0.003954754	0.054043918
Stard9	protein_coding	747.63162	-0.092349253	0.00415184	0.079536183
Gns	protein_coding	2455.041787	-0.092815244	0.002114838	0.034571024
Rin2	protein_coding	648.8451212	-0.092979124	0.005514319	0.094603974
Phkb	protein_coding	857.9621177	-0.094026831	0.003324993	0.080314409
Chchd10	protein_coding	1954.622282	-0.094112228	0.002223207	0.034875628
AW549877	protein_coding	1546.335108	-0.094258933	0.001128649	0.034337316
Dnajb2	protein_coding	2115.431168	-0.094652606	0.006454196	0.072589987
Entpd5	protein_coding	854.0337807	-0.095367683	0.00430917	0.07215724
Ankrd40	protein_coding	2837.600906	-0.0959498	3.42E-04	0.010157754
Eml1	protein_coding	1333.933386	-0.096202804	0.00592725	0.088243299
Tceal6	protein_coding	1627.32154	-0.096344386	0.005149963	0.079619679
Gm44250	TEC	95.27843299	-0.096404438	0.00141971	0.057434985
Gm29216	unprocessed_pseudogene	241951.4246	-0.096555785	0.002084531	0.034443487
Abat	protein_coding	6285.906506	-0.096604072	0.001560004	0.03595324
Ano10	protein_coding	415.7669938	-0.096878176	0.004049493	0.077974378
Prxl2a	protein_coding	2750.079896	-0.097684009	0.002177988	0.034571024
Synj2	protein_coding	2399.850407	-0.097751706	0.0060933	0.070039799

Tars	protein_coding	719.7148182	-0.097912282	0.001469388	0.066207193
Fam177a2	protein_coding	889.4814184	-0.098077719	0.004148821	0.092325026
Zcchc24	protein_coding	1934.593751	-0.098977334	0.004539313	0.074568921
Nbas	protein_coding	1242.121416	-0.099139728	0.003200677	0.077327286
Tmco3	protein_coding	811.9758263	-0.099245541	0.001407117	0.033904643
Slc35f6	protein_coding	720.2808391	-0.099305249	0.001528227	0.039970066
Pde4b	protein_coding	1807.358369	-0.099524211	0.004066215	0.054846462
Cln8	protein_coding	716.7640833	-0.100154647	0.002912835	0.099535834
Prelid3b	protein_coding	846.0306906	-0.100735531	9.80E-04	0.026215514
Gng12	protein_coding	1377.742281	-0.100817234	0.00291861	0.054846462
Atp1b3	protein_coding	2026.100201	-0.100977451	0.005641264	0.085777226
Firre	processed_transcript	2375.887242	-0.102041235	0.001523595	0.026215514
Mif	protein_coding	1738.880163	-0.102618862	0.002482995	0.066266398
Gm2808	unprocessed_pseudogene	909.66441	-0.102865902	0.001580492	0.036224532
Nfasc	protein_coding	9974.434817	-0.103677404	7.42E-04	0.014334598
Gm13339	unprocessed_pseudogene	10087.764	-0.103719064	0.002693647	0.027959497
Pkn1	protein_coding	1565.195203	-0.103856958	0.001202936	0.023707644
Hsd17b12	protein_coding	1311.537567	-0.104098518	0.00108446	0.036775551
Inpp5d	protein_coding	290.4376697	-0.104610266	0.004320852	0.094382875
Gpr137b	protein_coding	341.9895799	-0.104971861	0.002677008	0.080314409
Lamtor5	protein_coding	551.4235434	-0.10540022	0.002088518	0.080653045
Gm13340	unprocessed_pseudogene	82114.42273	-0.106219919	0.001315487	0.032558743
Snx6	protein_coding	720.6652378	-0.106312662	8.16E-04	0.043489531
mt-Co1	protein_coding	251305.4076	-0.106641524	0.002076465	0.023369837
Zfp944	protein_coding	297.7773646	-0.106953257	0.003002385	0.075050158
Ttll5	protein_coding	980.8963221	-0.107669827	0.00304338	0.073945425
Fryl	protein_coding	2234.980834	-0.108000325	2.48E-04	0.007915869
Tbccd1	protein_coding	310.9839935	-0.108362234	0.00360581	0.094946509
Abca2	protein_coding	9821.312598	-0.108427644	0.001185459	0.018259614
Wbp1	protein_coding	668.0467881	-0.108490374	9.59E-04	0.029926077
Taldo1	protein_coding	1921.132312	-0.109120655	8.24E-04	0.018901727
Tmem168	protein_coding	362.3120788	-0.109531902	0.001785903	0.061271064
Mettl7a1	protein_coding	687.9804869	-0.110032553	9.80E-04	0.04088789
Ptprd	protein_coding	4643.297762	-0.110228606	0.005230126	0.04379815
Cilk1	protein_coding	715.1801192	-0.110634917	0.002040842	0.07983599
Pnpo	protein_coding	894.9012412	-0.110937345	0.00118537	0.039867882
Slc35e3	protein_coding	708.3343807	-0.111389794	5.20E-04	0.032228305
Rnase4	protein_coding	275.6128151	-0.111860831	0.004434169	0.094005136
Scd1	protein_coding	4177.147265	-0.111862558	0.00643889	0.056476472
Tbc1d19	protein_coding	654.61359	-0.111975887	6.29E-04	0.036054247
C1qa	protein_coding	1015.403262	-0.112114716	0.002430317	0.066266398
Myo6	protein_coding	3270.911422	-0.112624814	7.30E-04	0.021965581
Endod1	protein_coding	2312.719206	-0.112680249	0.002781135	0.05435142
Dip2b	protein_coding	1962.184817	-0.113190662	2.11E-04	0.006851022
Fut10	protein_coding	209.2459095	-0.113198452	0.004429078	0.095372544
Tpt1	protein_coding	5053.064634	-0.113247699	8.85E-04	0.014615689
Pbxip1	protein_coding	1765.51502	-0.113565679	0.004132031	0.090667756
Ccdc190	protein_coding	351.2046309	-0.11377915	0.004140701	0.079424364
Cript	protein_coding	728.0156933	-0.114704851	4.96E-04	0.019119737
Scaper	protein_coding	782.3532482	-0.114759367	3.45E-04	0.024099372

Gm10925	unprocessed_pseudogene	256032.1334	-0.115857217	3.68E-04	0.007915869
Slc24a5	protein_coding	17.39128329	-0.115931637	7.33E-04	0.093568445
Fgfrl1	protein_coding	444.4000004	-0.116509767	0.00226131	0.072638065
Map4k4	protein_coding	3691.758813	-0.116781291	3.39E-04	0.006815959
Gm20300	lincRNA	903.6677805	-0.116902765	0.002750395	0.054004608
Nde1	protein_coding	595.7670151	-0.117316069	0.002813344	0.083376277
Imp4	protein_coding	674.9885822	-0.117467362	5.50E-04	0.027946207
Gtf3c6	protein_coding	543.2765368	-0.117498821	5.28E-04	0.032558743
Nek7	protein_coding	995.1877209	-0.117532796	0.001631603	0.036245621
Hadhb	protein_coding	956.3855119	-0.117701269	7.78E-04	0.022160155
mt-Nd4	protein_coding	159170.4768	-0.118714203	2.64E-04	0.006317644
Wdsub1	protein_coding	248.4348488	-0.120478717	0.002695309	0.070039799
Agpat4	protein_coding	1431.036804	-0.121010955	2.46E-04	0.012474892
Stard3	protein_coding	667.9161756	-0.12102581	3.83E-04	0.025740064
Fam234a	protein_coding	515.3713106	-0.121113024	0.003289601	0.088193226
Sirt2	protein_coding	3594.788332	-0.121118603	0.001385044	0.021892866
Gm11407	processed_pseudogene	1664.684858	-0.121323666	0.003181074	0.058243586
Sema4d	protein_coding	1229.123377	-0.122782698	8.15E-05	0.00636254
Rab22a	protein_coding	1311.332553	-0.122853592	0.001357722	0.038565794
Prlr	protein_coding	333.6835176	-0.123601685	6.38E-04	0.025695732
Hip1	protein_coding	1368.467237	-0.124415803	8.73E-04	0.019422714
Npy	protein_coding	859.5178123	-0.12445039	0.005092004	0.080314409
Ctsb	protein_coding	11701.68946	-0.124675271	8.04E-06	4.76E-04
Hsp90b1	protein_coding	6835.386856	-0.124781691	2.88E-04	0.009036481
Aldh3a2	protein_coding	1166.497726	-0.124825653	2.98E-04	0.011929873
Hexb	protein_coding	1874.023475	-0.124880011	8.78E-04	0.02444118
Gm12481	processed_pseudogene	926.9761631	-0.125201251	8.73E-05	0.005064105
Slc29a3	protein_coding	507.8873142	-0.125936515	8.78E-04	0.036186305
mt-Rnr2	Mt_rRNA	83523.71459	-0.126096141	0.00114202	0.017783307
Fgf1	protein_coding	1241.598944	-0.127114653	0.001689599	0.03791753
Adam19	protein_coding	477.2826562	-0.127156557	0.003067359	0.087586521
Vcan	protein_coding	506.7389042	-0.128017343	0.001858044	0.061493517
Septin4	protein_coding	4144.736396	-0.12829873	0.006146469	0.05467092
Rragb	protein_coding	1567.524078	-0.128465622	1.24E-04	0.006317644
Ctss	protein_coding	1153.96141	-0.129238246	0.001448961	0.033904643
Slc5a3	protein_coding	806.0334408	-0.129559463	0.004413952	0.095567348
Csf1r	protein_coding	2343.964549	-0.129838185	0.00220038	0.034646572
Enpp4	protein_coding	729.4763827	-0.130280226	0.00158593	0.041033279
Ctsd	protein_coding	4361.886989	-0.130623725	0.002016048	0.027536497
Trp53inp1	protein_coding	332.3935072	-0.130636056	0.001705587	0.050605054
Pip4k2a	protein_coding	2167.655032	-0.130664721	0.001038354	0.020859707
Mien1	protein_coding	776.4756279	-0.131819836	2.62E-04	0.012474892
Kif16b	protein_coding	357.2424734	-0.132022935	0.001372202	0.05180024
Gm4617	processed_pseudogene	277.71431	-0.132238	0.003476872	0.07055518
Aspscr1	protein_coding	704.9367767	-0.132684284	3.67E-04	0.025072404
Arhgap23	protein_coding	3768.369242	-0.133750582	7.98E-05	0.002698266
Kif13b	protein_coding	1821.118483	-0.133777536	0.002708631	0.069874667
Zdhhc9	protein_coding	1276.013051	-0.133911332	1.82E-04	0.010084286
Cers2	protein_coding	1429.517284	-0.134256326	0.003617712	0.08319895
Gm13341	unprocessed_pseudogene	5223.489329	-0.134647437	7.88E-05	0.004818884

mt-Cytb	protein_coding	176780.7022	-0.134889927	2.24E-04	0.005626474
Serinc5	protein_coding	2301.538533	-0.134928045	0.001143599	0.022901739
Phactr4	protein_coding	263.7017617	-0.135837292	0.003460443	0.080314409
Pcdhga2	protein_coding	244.2451027	-0.136178651	0.005387262	0.093284171
Cd164	protein_coding	1379.989894	-0.136598643	0.002776812	0.070464009
Slc39a11	protein_coding	442.5016627	-0.137168632	0.001641316	0.058407536
Lipa	protein_coding	455.3333202	-0.137743052	0.001710717	0.060333671
Zfp709	protein_coding	162.5616981	-0.137973768	0.002900594	0.082759602
Nmral1	protein_coding	292.5031024	-0.138440319	0.0040611	0.090684893
Arl2	protein_coding	863.1787793	-0.138779194	3.16E-04	0.016289483
Laptm4b	protein_coding	1713.706889	-0.138942436	2.47E-04	0.010157754
Kif5b	protein_coding	5269.298755	-0.139080979	7.43E-05	0.004657687
Col8a1	protein_coding	136.1840617	-0.139116851	5.27E-04	0.040173328
Golga7	protein_coding	1185.300264	-0.139341666	5.44E-04	0.023707644
Carhsp1	protein_coding	1075.894753	-0.139731396	0.003563994	0.064682926
2810004N23Rik	protein_coding	332.9146669	-0.139733862	4.48E-04	0.021109934
Dcaf8	protein_coding	3733.36741	-0.139815046	7.49E-07	9.76E-05
Ctsl	protein_coding	1468.43013	-0.139932248	0.001310949	0.042156648
Itgb5	protein_coding	1120.141958	-0.141627306	4.52E-04	0.020859707
Npc2	protein_coding	1153.222379	-0.142426543	0.00283245	0.077488587
Gpr34	protein_coding	214.5357907	-0.142991488	0.003556713	0.071808498
Atp11a	protein_coding	2057.542782	-0.143663434	0.00183865	0.032228305
Rpe	protein_coding	494.0748958	-0.14500002	7.64E-04	0.034571024
Fes	protein_coding	93.3019434	-0.146430866	0.003017924	0.077974378
Cln3	protein_coding	338.1522207	-0.146947688	8.91E-04	0.027998238
Sec11c	protein_coding	997.7412172	-0.147453429	3.84E-04	0.019083237
AU041133	protein_coding	97.8562297	-0.147877881	0.003278089	0.097490274
Jam3	protein_coding	782.7447569	-0.148384754	7.01E-04	0.038632639
Tmed10	protein_coding	901.9863166	-0.148647018	6.30E-04	0.026118132
Ttll7	protein_coding	5698.225933	-0.148862082	1.79E-04	0.004485209
Kif5a	protein_coding	66941.3139	-0.149056198	0.001122642	0.019026719
Taf13	protein_coding	354.6309005	-0.149197186	9.23E-04	0.038666456
Samd4	protein_coding	654.7840337	-0.149210689	8.17E-04	0.026215514
Limd1	protein_coding	296.8286424	-0.149576319	0.002337678	0.064682926
Ypel2	protein_coding	943.2211992	-0.149699432	0.002057108	0.063973951
Gm28661	unprocessed_pseudogene	189169.4183	-0.149811285	2.81E-05	0.001198992
Tmem126b	protein_coding	732.5745336	-0.149968558	1.53E-04	0.014176547
Car14	protein_coding	248.5141509	-0.150333569	0.002043535	0.066385105
Abcc9	protein_coding	175.7622496	-0.15174873	0.003410435	0.091761093
Urod	protein_coding	879.1847582	-0.152014602	9.32E-06	9.45E-04
Lrpprc	protein_coding	1653.133546	-0.153382149	9.83E-07	1.22E-04
Tmem229a	protein_coding	1366.092714	-0.153748863	9.51E-04	0.031129698
Pus1	protein_coding	287.2690416	-0.153881632	4.93E-04	0.022160155
Tpt1-ps6	processed_pseudogene	359.1623951	-0.154617167	4.66E-04	0.024885831
Pcdhga8	protein_coding	271.3569232	-0.155247969	0.003868876	0.088179348
Arhgap11a	protein_coding	84.89109188	-0.155743673	0.003492668	0.093284171
Arpc1b	protein_coding	307.1882031	-0.15593224	0.001227434	0.046848325
Nr6a1	protein_coding	268.5293236	-0.156172634	9.44E-04	0.034337316
Arhgap22	protein_coding	143.4466029	-0.156293425	0.002226499	0.066385105
Cyp27a1	protein_coding	97.06497964	-0.156379124	0.003599993	0.087419864

Adamtsl1	protein_coding	240.454659	-0.156475791	0.004079135	0.090827842
Mpp7	protein_coding	76.48973903	-0.157208168	0.002920824	0.08319895
Sox8	protein_coding	1031.932231	-0.15831156	5.44E-04	0.022989781
Ddo	protein_coding	219.9991999	-0.158441973	0.002591857	0.058387545
Cep162	protein_coding	479.9578555	-0.158896423	4.39E-04	0.023885383
Anapc13	protein_coding	321.2068123	-0.159370641	8.07E-04	0.031448379
Eif5a2	protein_coding	1103.964124	-0.159412514	4.11E-04	0.014567343
Rcbtb1	protein_coding	1086.536336	-0.159586226	0.001348137	0.043807871
Slc6a9	protein_coding	1235.829438	-0.160270219	0.002947113	0.055210896
Elovl7	protein_coding	392.2496444	-0.160820411	0.002304015	0.070227852
Lhpp	protein_coding	404.5164422	-0.160948101	3.39E-04	0.019422714
Adamtsl4	protein_coding	252.0376625	-0.16095869	0.003891839	0.076013435
Ufc1	protein_coding	1082.147173	-0.163160106	1.46E-04	0.007218457
Arhgap27	protein_coding	321.2204876	-0.163313043	0.001860201	0.046137463
mt-Nd5	protein_coding	78307.49945	-0.163702228	5.88E-04	0.011060614
Pik3c2b	protein_coding	703.2570475	-0.164264818	3.49E-04	0.024308172
Drc1	protein_coding	262.7845901	-0.164332108	0.002022604	0.058243586
Tmem176b	protein_coding	510.1584502	-0.165548856	0.002267585	0.072742762
Rassf2	protein_coding	1556.678645	-0.166803047	0.001206227	0.023718713
mt-Nd1	protein_coding	128744.1967	-0.167038096	1.60E-05	0.00142629
Psat1	protein_coding	2467.484782	-0.167241255	3.76E-04	0.010157754
Septin7	protein_coding	6381.957631	-0.167425537	2.10E-05	0.001750051
Cdr2	protein_coding	233.628124	-0.168239612	0.001352797	0.043747933
Aqp1	protein_coding	143.1209886	-0.16825029	3.94E-04	0.021965581
Lgmn	protein_coding	2239.588972	-0.168419835	1.05E-04	0.005626474
Bcas1	protein_coding	4629.040712	-0.169688806	0.00328863	0.034872394
Glb1	protein_coding	470.9879891	-0.169694853	0.001941745	0.063973951
Abcc3	protein_coding	69.51379673	-0.170014894	0.00319825	0.080395691
1700047M11Rik	lincRNA	377.0650542	-0.17013079	0.003388685	0.089725795
Elovl5	protein_coding	1466.597239	-0.170226746	1.89E-04	0.010157754
Atp7a	protein_coding	139.0295604	-0.171813906	0.002799584	0.080653045
Npc1	protein_coding	1905.716736	-0.173172513	4.44E-04	0.012327052
Crybg3	protein_coding	175.258857	-0.17323281	0.00221324	0.070039799
Slc7a10	protein_coding	925.7320592	-0.173620994	0.001745579	0.053096161
Olig2	protein_coding	563.8770271	-0.173793727	7.55E-04	0.040249839
Elovl1	protein_coding	422.6477714	-0.174918688	4.62E-04	0.024692898
Prkd3	protein_coding	499.4662377	-0.175419827	9.82E-04	0.030469095
Zfp488	protein_coding	90.40160325	-0.175672669	0.002128452	0.064577078
Pnpla2	protein_coding	420.1286571	-0.175932861	2.67E-04	0.01664069
Foxn3	protein_coding	1135.531549	-0.176335494	5.11E-04	0.022160155
Fam57a	protein_coding	284.1365326	-0.176730603	3.81E-04	0.021718286
Phlpp1	protein_coding	3040.261875	-0.176898974	8.07E-09	1.89E-06
Gm9800	processed_pseudogene	448.8416363	-0.177930353	4.50E-04	0.023059173
Cln5	protein_coding	259.8691484	-0.178023877	6.78E-04	0.027315349
Efcab14	protein_coding	1268.103471	-0.178053292	9.56E-05	0.007076741
Qtrt1	protein_coding	316.9787044	-0.178781113	6.85E-04	0.027536497
Rps4l	transcribed_processed_pseu	347.9221189	-0.17914732	0.002804796	0.07983599
Litaf	protein_coding	470.9292126	-0.179396671	0.002863166	0.083376277
F5	protein_coding	417.996651	-0.180440854	3.46E-04	0.02030074
Atp10b	protein_coding	75.24321708	-0.181599208	0.001979876	0.066218066

Rnf13	protein_coding	2223.41254	-0.181993423	5.18E-04	0.012857381
Degs1	protein_coding	1555.596991	-0.182238867	7.10E-05	0.00348794
Pfdn2	protein_coding	1575.617247	-0.182944559	6.48E-05	0.005597234
Kcna6	protein_coding	2238.272778	-0.183536743	3.28E-04	0.009331775
Ddc	protein_coding	108.360007	-0.184553681	0.002455176	0.070039363
Hey2	protein_coding	87.94702866	-0.184588409	0.002774478	0.080395691
Ptpdc1	protein_coding	852.0122729	-0.185563863	6.90E-06	0.001027694
Itprid2	protein_coding	1329.192875	-0.186622216	5.86E-04	0.01470785
Slc4a5	protein_coding	180.9423883	-0.18675367	3.09E-04	0.017812514
Vps26a	protein_coding	1143.780203	-0.187268887	5.05E-07	1.40E-04
Gpt	protein_coding	354.7335188	-0.187608386	0.001318412	0.04988858
Gba	protein_coding	795.4330184	-0.188019385	1.50E-05	0.002749764
Enpp2	protein_coding	12131.95698	-0.188130833	3.20E-04	0.012545809
Padi2	protein_coding	1061.701307	-0.188427805	6.34E-04	0.026215514
Syngr2	protein_coding	191.1519516	-0.189364643	0.001783512	0.076152581
Col27a1	protein_coding	254.2087176	-0.190556162	0.001171814	0.033904643
Unc5b	protein_coding	914.0567692	-0.191144119	0.002186087	0.04542821
Mest	protein_coding	330.6192517	-0.191368926	0.00248137	0.066385105
Sh3gl3	protein_coding	605.8697436	-0.191622026	2.00E-04	0.017111209
Tjp2	protein_coding	873.4086573	-0.191737342	4.15E-04	0.02028416
Pcx	protein_coding	2032.37868	-0.19191354	2.42E-06	3.26E-04
Micall1	protein_coding	2116.384893	-0.193562395	4.25E-04	0.011928592
Ccdc163	protein_coding	64.93885098	-0.193703782	0.002315787	0.066266398
Sema6a	protein_coding	741.2483565	-0.193862801	0.001697395	0.07055518
Tppp3	protein_coding	643.6476524	-0.194820472	0.001653969	0.070039799
Snx22	protein_coding	178.0482276	-0.194829447	0.001051056	0.066247407
Ctsa	protein_coding	2318.395924	-0.195273306	1.15E-05	8.47E-04
Acadl	protein_coding	695.7647033	-0.195589836	8.61E-05	0.009398095
Ccdc80	protein_coding	90.34767097	-0.196311105	0.002379158	0.073079718
Stra6	protein_coding	312.3495804	-0.197185427	0.002314004	0.064577078
Dusp19	protein_coding	232.7120835	-0.197481663	2.90E-04	0.015580081
Olfml1	protein_coding	266.3577929	-0.199082292	0.001553784	0.04843957
Fcgr3	protein_coding	207.452969	-0.199411754	0.001883423	0.05435142
Aldh2	protein_coding	1998.338044	-0.199539275	1.41E-04	0.006864643
Tmcc3	protein_coding	1535.860257	-0.200509773	5.22E-04	0.017473871
Pllp	protein_coding	1159.949046	-0.20068161	0.001712372	0.038262506
Plekhg3	protein_coding	592.8256737	-0.201218638	0.002035925	0.068595059
Klhl4	protein_coding	251.433341	-0.201775597	0.00193705	0.055178179
Mob3b	protein_coding	142.6150242	-0.201898391	0.002202498	0.066266398
Hps3	protein_coding	368.211414	-0.202611969	4.67E-05	0.003835067
Acy1	protein_coding	160.4378602	-0.203272017	9.83E-04	0.051651046
Apln	protein_coding	483.4475275	-0.203387041	0.001457042	0.038632639
Gm28437	unprocessed_pseudogene	186606.8958	-0.203491432	8.96E-06	7.14E-04
Cdc42ep2	protein_coding	227.512154	-0.203817897	0.001767474	0.053096161
Trp53inp2	protein_coding	4323.847464	-0.20499823	6.47E-05	0.002009971
Cldn2	protein_coding	126.7531542	-0.205823865	2.96E-04	0.022542628
Usp54	protein_coding	2027.587559	-0.20615035	1.87E-04	0.008554616
Hapln2	protein_coding	371.4623235	-0.206406615	0.002083794	0.068783018
Zfp367	protein_coding	379.6086005	-0.207256973	2.80E-04	0.01735962
Tfr2	protein_coding	177.4720462	-0.207408182	0.001191421	0.07055518

Tgfbi	protein_coding	94.34993503	-0.207730578	0.001360867	0.050605054
Scly	protein_coding	323.4190446	-0.207858219	1.97E-04	0.010157754
Cdc37l1	protein_coding	3067.044982	-0.207966886	5.36E-06	6.30E-04
Erbin	protein_coding	2280.208147	-0.209961783	0.001122557	0.021892866
Pla2g4a	protein_coding	62.58934595	-0.210596424	0.001991151	0.066247407
Rasgrp3	protein_coding	318.281473	-0.21110159	0.001077888	0.037261656
Steap1	protein_coding	24.26144251	-0.211862048	2.83E-04	0.05055761
Cndp2	protein_coding	1077.766432	-0.21244448	1.87E-06	3.81E-04
Csrp1	protein_coding	5899.530859	-0.212495162	8.40E-04	0.01550213
Zfp783	transcribed_unprocessed_p	184.3488413	-0.21292727	0.001422461	0.066266398
Clcn5	protein_coding	272.954577	-0.21596152	6.71E-04	0.031613303
S100b	protein_coding	2314.042683	-0.216232463	2.72E-04	0.010952236
Dctd	protein_coding	135.567243	-0.21732008	0.001515264	0.069012171
Creb3l2	protein_coding	496.0555316	-0.220932133	1.30E-04	0.01008659
Wipf1	protein_coding	348.5432793	-0.222718646	6.07E-04	0.029342753
Plekhb1	protein_coding	8004.371621	-0.222830793	0.001179456	0.023369837
Daam2	protein_coding	1412.03411	-0.223541864	6.73E-05	0.005626474
Tpd52	protein_coding	993.2331758	-0.223698878	2.09E-05	0.002449799
Kif13a	protein_coding	1028.075752	-0.223874693	9.91E-05	0.007407308
Mfsd1	protein_coding	565.6757688	-0.224071145	1.81E-06	3.05E-04
Trim36	protein_coding	190.6200229	-0.2246068	0.001091211	0.040179362
Copa	protein_coding	5196.710659	-0.225787192	1.31E-12	4.97E-10
Cpm	protein_coding	333.5604964	-0.227781094	0.001227221	0.034571024
Pigm	protein_coding	338.6617518	-0.227924105	3.86E-05	0.003835067
Heatr5a	protein_coding	283.6112852	-0.228007931	6.51E-05	0.005626474
Ncf1	protein_coding	144.4558563	-0.22882361	7.18E-04	0.032741176
Mtmr10	protein_coding	572.7905356	-0.229703732	1.52E-04	0.014176547
Wnk1	protein_coding	6645.719185	-0.229929714	1.68E-06	1.85E-04
Selplg	protein_coding	655.8143165	-0.229958233	5.66E-04	0.033930701
Mtus1	protein_coding	806.2851911	-0.230452902	5.05E-04	0.022160155
Ldlrap1	protein_coding	98.76637771	-0.232097801	0.001419546	0.052157326
Gm6483	unprocessed_pseudogene	146.4822795	-0.232364916	7.35E-04	0.033277079
Adam17	protein_coding	403.1326784	-0.23285104	8.18E-06	0.001395063
Smpdl3a	protein_coding	469.3144198	-0.234011503	3.87E-04	0.016289483
Anln	protein_coding	888.3566566	-0.234032557	0.001460094	0.046348009
Cuta	protein_coding	626.9303825	-0.234489395	4.27E-07	1.39E-04
Dip2a	protein_coding	2286.131087	-0.237064773	1.61E-05	0.001008181
Bloc1s1	protein_coding	612.6027938	-0.237740666	8.56E-05	0.005626474
Sec14l5	protein_coding	778.244679	-0.238658008	0.001228864	0.034571024
Gm14419	protein_coding	352.9410027	-0.239355102	1.92E-04	0.013835724
Clic4	protein_coding	1786.820062	-0.240305917	9.53E-04	0.034160063
Nat8f6	protein_coding	29.89995977	-0.241119592	0.001292942	0.088935864
Mrm1	protein_coding	195.943812	-0.243173334	4.72E-04	0.024433424
Wipi1	protein_coding	555.0715456	-0.24384252	4.75E-06	9.39E-04
Qk	protein_coding	6374.464532	-0.246618877	4.10E-06	5.16E-04
Cdh19	protein_coding	147.1440972	-0.248034753	0.001156559	0.05772608
Bfsp2	protein_coding	125.8278103	-0.249491779	0.001130752	0.068788747
Ppp1r14b	protein_coding	272.9650947	-0.249598009	6.83E-05	0.005626474
Mcam	protein_coding	647.3135487	-0.250078011	8.77E-04	0.045099785
Evi2a	protein_coding	486.860173	-0.250108772	0.001080012	0.04357186

Rab31	protein_coding	1622.998107	-0.250244122	2.27E-06	3.81E-04
Zfp101	protein_coding	242.6776282	-0.250416094	5.53E-05	0.004271214
Bex4	protein_coding	164.2629642	-0.250673495	1.73E-04	0.012545809
Magt1	protein_coding	375.8268921	-0.250744953	4.37E-04	0.022621493
4930456G14Rik	TEC	15.46786643	-0.250796625	6.28E-04	0.087586521
mt-Nd2	protein_coding	82317.13341	-0.25083871	1.13E-07	2.67E-05
Olig1	protein_coding	2124.71593	-0.251801227	6.89E-05	0.003423672
Omg	protein_coding	2254.151145	-0.252345718	2.74E-07	5.99E-05
Dock1	protein_coding	851.7945689	-0.252973248	2.93E-05	0.003095468
Gm21092	unprocessed_pseudogene	122.0067741	-0.253133952	5.62E-04	0.027536497
Prdm16	protein_coding	415.5026586	-0.255628568	7.36E-04	0.033277079
Ccdc91	protein_coding	779.4963994	-0.256878911	2.30E-06	3.71E-04
Gpr37	protein_coding	1942.725148	-0.257025066	7.84E-04	0.022206733
Gm28438	unprocessed_pseudogene	1438.599554	-0.257203704	7.48E-05	0.005988712
Gm5884	processed_pseudogene	88.01519611	-0.259380726	5.28E-04	0.029276474
Hcls1	protein_coding	104.114124	-0.259698204	7.16E-04	0.032741176
Dusp23	protein_coding	122.7477678	-0.262131981	2.97E-04	0.018498459
mt-Nd3	protein_coding	16223.08776	-0.262170048	1.30E-08	2.16E-06
Arap1	protein_coding	474.3527846	-0.263176677	2.93E-05	0.00364464
Gm14403	protein_coding	128.8361416	-0.265370617	1.76E-04	0.01269459
Taf6l	protein_coding	239.5516748	-0.266157377	4.03E-05	0.00394183
Lactb2	protein_coding	277.1440973	-0.267345463	9.62E-05	0.008306622
Dusp15	protein_coding	301.409645	-0.268098303	6.18E-04	0.022160155
Gamt	protein_coding	205.9569363	-0.268718759	1.84E-04	0.011434214
Metap1d	protein_coding	424.6604177	-0.269545502	3.65E-07	1.24E-04
H2bc6	protein_coding	75.78405527	-0.2704798	7.54E-04	0.033904643
Plaat3	protein_coding	804.8979665	-0.271884075	3.47E-04	0.017547411
Slc44a1	protein_coding	2224.461287	-0.274361941	9.56E-05	0.004271214
Tac1	protein_coding	134.818696	-0.27465066	8.74E-04	0.047194071
Tmbim1	protein_coding	816.3110509	-0.275126416	5.64E-04	0.025944587
Efhd1	protein_coding	527.4517743	-0.276586687	6.56E-04	0.030466724
Cmtm5	protein_coding	871.841792	-0.277525498	2.37E-05	0.002902382
Coprs	protein_coding	318.065925	-0.278392585	1.19E-04	0.008344449
Tmem126a	protein_coding	307.1393388	-0.279162498	2.68E-06	6.07E-04
Pld4	protein_coding	203.5630327	-0.279182642	8.18E-05	0.007385162
Selenop	protein_coding	4161.524432	-0.280563955	2.02E-04	0.004863344
Sh3tc2	protein_coding	82.47524788	-0.280770801	7.58E-04	0.031905574
Arsa	protein_coding	794.9558078	-0.281293276	4.70E-08	2.68E-05
Gng11	protein_coding	162.8024775	-0.283525262	7.58E-04	0.032256828
Kat2b	protein_coding	503.4393461	-0.285253086	3.79E-04	0.021892866
Orai3	protein_coding	599.2478923	-0.285870911	1.53E-06	4.92E-04
Itgb4	protein_coding	367.6359673	-0.286560712	7.99E-04	0.025955808
Gpr17	protein_coding	1459.852102	-0.287858791	5.61E-05	0.004520822
Phldb1	protein_coding	4338.450296	-0.28855717	1.96E-04	0.004778571
Josd2	protein_coding	730.4248719	-0.289446215	6.69E-05	0.004863344
Smpdl3b	protein_coding	36.92480443	-0.2900351	7.36E-04	0.062310475
Zfp781	protein_coding	258.6716469	-0.290574889	9.29E-05	0.006864643
Gm3365	unprocessed_pseudogene	133.4819842	-0.294563703	2.57E-04	0.016622814
Fbxo32	protein_coding	461.1081367	-0.294608383	2.07E-04	0.014567343
Wfdc2	protein_coding	28.52976218	-0.295123351	1.74E-04	0.036682738

Rufy1	protein_coding	833.2003705	-0.295620899	3.06E-10	2.18E-07
Washc5	protein_coding	937.8547816	-0.296611443	8.82E-14	1.16E-10
Chst3	protein_coding	107.9230779	-0.297447379	3.94E-04	0.021965581
Gyg	protein_coding	592.3440985	-0.298133908	1.62E-06	2.79E-04
Gusb	protein_coding	268.468105	-0.29838078	1.76E-04	0.011093825
Sox2ot	processed_transcript	816.3021113	-0.29859888	3.69E-04	0.019927819
Carns1	protein_coding	211.7496683	-0.299952692	4.91E-04	0.022160155
Sdf2l1	protein_coding	181.9043809	-0.30009116	1.64E-04	0.015340988
Nit1	protein_coding	737.1468806	-0.301452117	8.83E-10	9.12E-07
Gab1	protein_coding	948.9028429	-0.301851621	2.06E-04	0.009036481
Atic	protein_coding	577.4734062	-0.301962396	7.80E-09	5.25E-06
Prrg1	protein_coding	127.4266621	-0.30283449	5.48E-04	0.034337316
Ppox	protein_coding	568.8050301	-0.304797621	3.97E-09	3.00E-06
Gna12	protein_coding	2813.758405	-0.306589574	4.23E-07	5.99E-05
9330117O12Rik	lincRNA	41.07673176	-0.307222909	6.42E-04	0.056664738
Slc2a5	protein_coding	103.8524068	-0.309585538	2.41E-04	0.017547411
Map3k19	protein_coding	144.9500501	-0.310662506	5.02E-04	0.032613413
2600014E21Rik	lincRNA	52.80970225	-0.310859321	5.68E-04	0.077792794
Fcer1g	protein_coding	135.6084631	-0.311345543	3.82E-04	0.020416143
Arsg	protein_coding	638.3745661	-0.312261974	5.88E-04	0.034405321
C030029H02Rik	lincRNA	92.02877594	-0.314411036	5.54E-04	0.024692898
Pdlim2	protein_coding	262.987933	-0.31480822	5.59E-04	0.023885383
Gpr183	protein_coding	18.73475891	-0.315936703	5.00E-04	0.073543123
Cntn2	protein_coding	4265.701869	-0.317796262	7.74E-05	0.002649867
Arrdc3	protein_coding	1097.477104	-0.317927881	2.96E-09	1.83E-06
Pacs2	protein_coding	4061.9028	-0.318447574	1.52E-05	0.001383553
Commd8	protein_coding	836.2861688	-0.323439392	9.84E-12	9.91E-09
Rmnd1	protein_coding	432.3668956	-0.32402114	6.45E-07	1.34E-04
Pls1	protein_coding	323.751671	-0.325181613	3.88E-04	0.018883964
Mag	protein_coding	4775.666261	-0.327512975	5.11E-04	0.00908892
Wnk3	protein_coding	581.9061199	-0.328005292	2.28E-06	6.61E-04
Ptgs1	protein_coding	277.172316	-0.328218956	1.66E-05	0.001928426
Tom1	protein_coding	774.8564694	-0.329710024	7.80E-06	0.001644352
Tcn2	protein_coding	464.7945141	-0.330056221	4.93E-04	0.019083237
Slc25a13	protein_coding	48.4731268	-0.331504726	3.68E-04	0.03791753
Renbp	protein_coding	40.87603312	-0.3325572	4.83E-04	0.054846462
Rffl	protein_coding	352.6793761	-0.336889107	6.63E-05	0.006400706
Gm21811	unprocessed_pseudogene	254.7180369	-0.337816591	1.45E-05	0.001796797
Prr5l	protein_coding	303.8402585	-0.33810313	2.82E-04	0.017812514
Acot6	protein_coding	195.5424532	-0.340305593	4.14E-06	7.82E-04
Fuca2	protein_coding	697.8071374	-0.340910242	1.54E-07	7.77E-05
Serpinb1a	protein_coding	187.1755921	-0.340963873	4.20E-04	0.022542628
Mmp2	protein_coding	62.60808111	-0.341184205	4.38E-04	0.025457554
Pcdhga7	protein_coding	243.8020024	-0.341725751	5.54E-05	0.005626474
6820431F20Rik	transcribed_unprocessed_p	1288.762717	-0.342762539	5.84E-07	8.05E-05
Trim25	protein_coding	133.9501401	-0.342829753	2.83E-04	0.022160155
Cyp2j12	protein_coding	67.99475483	-0.34387881	3.60E-04	0.018977495
Qdpr	protein_coding	4033.76131	-0.344340273	1.47E-04	0.004772756
Rhog	protein_coding	540.6920437	-0.344343047	2.41E-04	0.016289483
Ttyh2	protein_coding	1122.937373	-0.349370101	2.82E-04	0.011191311

Lap3	protein_coding	1076.318231	-0.349678486	6.99E-08	1.74E-05
Pde1c	protein_coding	199.0757137	-0.350687647	2.16E-04	0.014567343
Osgep	protein_coding	463.1635222	-0.351168822	4.25E-09	1.89E-06
Rnf122	protein_coding	145.1936445	-0.352167967	6.88E-05	0.010157754
Cerox1	processed_transcript	542.7184552	-0.355115825	2.53E-05	0.004082073
Gal3st1	protein_coding	237.8745327	-0.359014817	1.58E-04	0.010157754
Lims2	protein_coding	240.7053732	-0.359146361	4.06E-06	7.14E-04
Rab3il1	protein_coding	297.7112402	-0.359245159	1.01E-05	0.001618388
Bmp2k	protein_coding	433.9149754	-0.362831368	2.84E-06	6.32E-04
Kank1	protein_coding	523.8170719	-0.362971846	7.14E-06	8.99E-04
Col6a4	protein_coding	152.0912234	-0.363241447	1.21E-04	0.01516501
A330049N07Rik	lincRNA	27.13648273	-0.363273563	2.84E-04	0.050605054
Nipal4	protein_coding	120.0630554	-0.363646275	0.000346537	0.025072404
Tns3	protein_coding	1993.343259	-0.363877081	1.24E-10	3.97E-08
Gm5512	transcribed_unprocessed_p	179.9553085	-0.365700758	1.13E-05	0.001750051
Tmem98	protein_coding	235.4758707	-0.365813908	3.20E-04	0.014332133
Plin3	protein_coding	371.1952631	-0.367283779	7.54E-05	0.006982479
Ermn	protein_coding	1485.713088	-0.367556508	3.32E-04	0.010084286
Slc38a7	protein_coding	837.1799316	-0.36899526	2.69E-08	6.93E-06
Grm3	protein_coding	868.1797412	-0.370925593	1.23E-05	0.001586297
Ccp110	protein_coding	1887.275719	-0.372706298	1.22E-05	0.001164893
Prdx4	protein_coding	149.649554	-0.37565066	8.90E-05	0.00785131
Myrf	protein_coding	2161.921027	-0.377559903	1.11E-04	0.004778571
Prr18	protein_coding	1323.018554	-0.378618647	2.53E-04	0.014176547
Gatm	protein_coding	2560.396735	-0.379068518	1.50E-04	0.005626474
Wwp2	protein_coding	961.6201111	-0.379535942	1.94E-14	3.42E-11
Tmem63a	protein_coding	953.6076906	-0.380636907	2.01E-04	0.008837417
Stat5a	protein_coding	68.64773933	-0.380778879	1.12E-04	0.008344449
Sox10	protein_coding	1336.628583	-0.385685874	5.26E-05	0.00364464
Tmem72	protein_coding	244.0656235	-0.386877713	9.80E-05	0.006246486
Dhtkd1	protein_coding	210.3761672	-0.38733081	2.37E-06	5.49E-04
Ppp1r14a	protein_coding	209.5901838	-0.390706075	2.12E-04	0.010679469
Hps1	protein_coding	387.6240473	-0.392563753	4.43E-09	1.89E-06
Hexa	protein_coding	1109.766973	-0.393255027	6.47E-12	4.48E-09
Gm10557	processed_pseudogene	356.3429193	-0.393836139	3.26E-06	4.98E-04
Idua	protein_coding	424.0042457	-0.394066241	5.60E-10	4.83E-07
Aga	protein_coding	99.25257985	-0.394691508	1.73E-04	0.012545809
G6pc3	protein_coding	864.0830045	-0.395059982	4.12E-09	2.08E-06
Ndrg1	protein_coding	2905.022413	-0.397575615	1.29E-04	0.005151251
Gltp	protein_coding	824.8374291	-0.398070659	1.65E-06	2.32E-04
Mbp	protein_coding	66102.06531	-0.401610307	1.49E-04	0.004778571
Myo1e	protein_coding	347.0938952	-0.402711261	2.24E-05	0.002902382
Mal	protein_coding	5223.124267	-0.404380552	2.21E-04	0.005151251
Upk1b	protein_coding	21.35237767	-0.405712165	1.47E-04	0.032256828
Pld1	protein_coding	186.4371306	-0.409359076	4.31E-05	0.007385162
Myo1d	protein_coding	512.89663	-0.414935449	9.62E-05	0.008554616
Manba	protein_coding	231.4187624	-0.415838194	1.20E-05	0.00179718
Sgk3	protein_coding	225.9285788	-0.417877675	9.52E-05	0.008245489
Gsn	protein_coding	1616.892321	-0.419017508	8.93E-05	0.006815959
Hipk2	protein_coding	1501.881246	-0.420930042	3.67E-07	5.80E-05

Tspan2	protein_coding	2748.68455	-0.421843723	8.61E-05	0.003877453
Gm30082	bidirectional_promoter_lncl	15.99769688	-0.421976559	1.42E-04	0.031981074
Gjc2	protein_coding	319.3337123	-0.422102171	6.53E-05	0.005585657
0610040J01Rik	protein_coding	83.73401783	-0.4225413	5.08E-05	0.005855133
Trim59	protein_coding	404.5173343	-0.423018269	1.04E-04	0.008554616
Cd82	protein_coding	500.8723184	-0.425583808	1.30E-04	0.01008659
Sspo	protein_coding	90.49178902	-0.425923035	1.59E-04	0.012941899
Dmp1	protein_coding	104.4915206	-0.42662788	1.57E-04	0.010848213
Ifi27	protein_coding	603.0915839	-0.427851429	7.24E-05	0.005151251
Gm19500	processed_transcript	106.5485768	-0.42795452	1.73E-04	0.012545809
Slc12a2	protein_coding	1937.066606	-0.428423943	1.11E-04	0.005945459
Catspere2	protein_coding	55.92874332	-0.429289234	1.53E-04	0.032376175
Olfml3	protein_coding	453.7817378	-0.431554311	2.75E-06	6.32E-04
Fgd3	protein_coding	265.366039	-0.432470105	2.45E-07	7.52E-05
Ppfibp2	protein_coding	307.0810312	-0.432628192	1.08E-04	0.006714139
Mcm3	protein_coding	68.08342319	-0.432820021	6.30E-05	0.006218393
Mrgpre	protein_coding	170.6193157	-0.43328627	5.88E-05	0.005891277
Gm48678	antisense	32.24520988	-0.434332909	1.29E-04	0.019083237
Gpx1	protein_coding	725.9521476	-0.437301441	1.94E-11	1.25E-08
Pogk	protein_coding	1487.383141	-0.438334205	7.72E-09	1.89E-06
Scd3	protein_coding	128.1144104	-0.441008612	1.13E-04	0.009268192
Plekhh1	protein_coding	1173.695178	-0.441689351	9.11E-05	0.006815959
Snx8	protein_coding	236.6988879	-0.441842597	2.02E-07	6.40E-05
Nkain1	protein_coding	435.1638555	-0.441973033	4.52E-06	8.87E-04
Insc	protein_coding	109.2043426	-0.444252422	1.15E-04	0.009382035
Tpp1	protein_coding	1722.446368	-0.449375539	6.71E-19	8.34E-16
Gjc3	protein_coding	1200.110391	-0.449391761	5.43E-05	0.003737477
Cnp	protein_coding	7432.599029	-0.451353999	5.97E-05	0.002106959
B3gnt9	protein_coding	74.43010232	-0.455048987	5.52E-05	0.005626474
Aldh3b1	protein_coding	100.6121032	-0.455104498	1.41E-05	0.001814091
BC024063	protein_coding	77.27915913	-0.459284926	4.86E-05	0.004863344
Folh1	protein_coding	131.8582721	-0.461728887	1.60E-05	0.00226941
Arhgef10	protein_coding	1320.283515	-0.46708204	6.85E-06	7.70E-04
Pecr	protein_coding	84.00739688	-0.467404528	1.26E-05	0.002081442
Ttr	protein_coding	17345.96407	-0.46980315	8.21E-05	0.003835067
Fgfr2	protein_coding	1242.038277	-0.470334407	8.70E-06	7.07E-04
Gjb1	protein_coding	289.9950255	-0.471327235	9.85E-05	0.007383203
Marchf8	protein_coding	1492.437187	-0.473330852	1.35E-10	6.46E-08
Chn2	protein_coding	628.5097005	-0.478826428	2.56E-07	1.14E-04
Il33	protein_coding	1150.84928	-0.479945556	2.70E-05	0.002902382
Aldh1l2	protein_coding	120.3223897	-0.487135525	2.32E-07	1.39E-04
Chdh	protein_coding	209.6006042	-0.48956904	8.80E-05	0.007828905
Gnpda1	protein_coding	336.7879525	-0.491142422	5.81E-15	1.49E-11
Aspa	protein_coding	362.1961394	-0.494233504	6.48E-05	0.006317644
Cyp2j6	protein_coding	406.4308739	-0.495078496	1.51E-06	3.68E-04
Plxnb3	protein_coding	980.0647074	-0.497367381	8.20E-07	2.09E-04
Hoga1	protein_coding	23.04933204	-0.499446576	7.86E-05	0.021047266
Pde8a	protein_coding	519.9124213	-0.506870767	1.26E-05	0.001905193
Tmem88b	protein_coding	1572.548067	-0.508294163	3.05E-05	0.001756385
Enpp6	protein_coding	240.5911662	-0.509774697	2.76E-05	0.002503209

Ogdhl	protein_coding	2398.141519	-0.511479259	1.37E-26	2.77E-23
Lpar1	protein_coding	811.3214476	-0.513997346	2.20E-05	0.001790297
Car2	protein_coding	4044.217197	-0.520497627	2.04E-07	1.98E-05
Mobp	protein_coding	5433.899048	-0.530471774	2.08E-05	9.53E-04
Tnfaip6	protein_coding	142.7887514	-0.53239633	3.35E-05	0.003835067
Cryab	protein_coding	2029.508201	-0.534024074	1.25E-06	1.40E-04
Cldn11	protein_coding	2719.184301	-0.539840288	1.02E-05	9.77E-04
Mog	protein_coding	1291.987519	-0.544540507	1.58E-05	0.001386054
Igf2r	protein_coding	1107.16326	-0.547242437	2.66E-18	4.02E-15
Gm38642	processed_transcript	333.6778017	-0.548660566	9.00E-06	0.001475981
Hhip	protein_coding	230.0065282	-0.552414098	6.27E-06	9.53E-04
Hmox1	protein_coding	105.8490811	-0.569510464	3.16E-07	9.76E-05
Mfrp	protein_coding	211.7730216	-0.573413015	6.55E-05	0.005626474
Zfp677	protein_coding	41.3277882	-0.580822951	3.20E-05	0.011184395
mt-Ti	Mt_tRNA	171.8728712	-0.584804591	1.27E-07	8.57E-05
Fa2h	protein_coding	954.9769458	-0.58832202	4.53E-06	5.59E-04
Plp1	protein_coding	34295.96924	-0.590744288	3.51E-06	2.09E-04
Smco3	protein_coding	185.4464783	-0.593226235	8.04E-07	2.09E-04
Trf	protein_coding	6871.520953	-0.593615668	5.49E-06	3.05E-04
Uap1l1	protein_coding	236.9263661	-0.60204491	1.05E-11	9.91E-09
Erbb3	protein_coding	403.8842731	-0.607048293	6.17E-07	1.85E-04
Ccl9	protein_coding	27.32864401	-0.611622847	1.48E-05	0.006786061
Shmt1	protein_coding	47.73992332	-0.612336409	1.75E-06	0.0010018
Prkcq	protein_coding	279.1140463	-0.61241417	2.16E-06	3.56E-04
Cfap47	protein_coding	28.7801248	-0.61372285	2.35E-05	0.005855133
Gaa	protein_coding	8818.266547	-0.614899307	1.43E-29	1.63E-26
Plin2	protein_coding	97.91852921	-0.618179801	3.06E-08	1.44E-05
F11r	protein_coding	140.6842021	-0.618281576	1.02E-05	0.001623746
Dock5	protein_coding	389.7044762	-0.618747696	7.71E-06	0.001312584
Rad9a	protein_coding	182.0755657	-0.624695245	1.23E-14	3.42E-11
Hpgd	protein_coding	91.40871733	-0.630108391	2.32E-07	8.39E-05
Abca8a	protein_coding	278.2943754	-0.639403404	1.11E-08	3.87E-06
Kcnk13	protein_coding	163.3067093	-0.640229864	1.08E-08	5.08E-06
Slc26a11	protein_coding	110.1367856	-0.649700561	1.19E-09	8.70E-07
Prune2	protein_coding	1380.971721	-0.649763324	8.26E-14	5.52E-11
Gm45847	lincRNA	59.75248724	-0.651322301	7.35E-07	2.09E-04
Tmem125	protein_coding	151.2018668	-0.664327032	1.54E-07	8.05E-05
9630013A20Rik	antisense	51.34647869	-0.68930361	1.32E-06	0.001081744
Adamts4	protein_coding	684.5928756	-0.695602967	6.45E-09	4.55E-06
Sgsh	protein_coding	187.7207662	-0.709231889	1.34E-11	1.25E-08
Ucp2	protein_coding	636.1772057	-0.750435426	3.79E-08	2.27E-05
Ugt8a	protein_coding	1169.881196	-0.7603561	4.87E-09	2.34E-06
Il17rb	protein_coding	35.04463295	-0.778995709	1.83E-06	8.87E-04
Dnph1	protein_coding	44.52947293	-0.793197519	7.35E-09	1.11E-05
Rnf17	protein_coding	9.626386383	-0.848149499	1.35E-05	0.023059173
Zfp951	protein_coding	46.09362316	-0.854708244	1.38E-09	3.49E-06
Slc45a3	protein_coding	21.752354	-0.96872511	7.17E-07	7.54E-04
Zfp459	protein_coding	21.87800897	-1.043455236	1.74E-07	2.30E-04
Zfp946	protein_coding	212.0446048	-1.058099692	1.56E-37	7.77E-34
Usf1	protein_coding	774.7391321	-3.317553704	4.89E-121	7.33E-117

Dpp7	protein_coding	324.9127972	-3.477722008	7.56E-111	7.20E-107
4930447C04Rik	protein_coding	85.09795122	-3.77072194	5.37E-31	2.78E-27
